# ATP insertion opposite 8-oxo-deoxyguanosine by Pol4 mediates error-free tolerance in *Schizosaccharomyces pombe*

**DOI:** 10.1093/nar/gku711

**Published:** 2014-08-08

**Authors:** Guillermo Sastre-Moreno, Arancha Sánchez, Verónica Esteban, Luis Blanco

**Affiliations:** Centro de Biología Molecular “Severo Ochoa” (CSIC-UAM), Universidad Autónoma, 28049 Madrid, Spain

## Abstract

7,8-Dihydro-8-oxo-deoxyguanosine (8oxodG) is a highly premutagenic DNA lesion due to its ability to mispair with adenine. *Schizosaccharomyces pombe* lacks homologs for relevant enzymes that repair 8oxodG, which suggests that this lesion could be persistent and must be tolerated. Here we show that *Sp*Pol4, the unique PolX in fission yeast, incorporates ATP opposite 8oxodG almost exclusively when all nucleotides (ribos and deoxys) are provided at physiological concentrations. Remarkably, this *Sp*Pol4-specific reaction could also occur during the NHEJ of DSBs. In cell extracts, misincorporation of ATP opposite 8oxodG was shown to be *Sp*Pol4-specific, although RNase H2 efficiently recognized the 8oxodG:AMP mispair to remove AMP and trigger error-free incorporation of dCTP. These data are the first evidence that ribonucleotides can be used safely for 8oxodG tolerance, suggesting that insertion of the highly abundant ATP substrate could be beneficial to promote efficient and error-free repair of 8oxodG-associated DSBs. Moreover, we demonstrate that purified *Sp*Pol4 uses 8oxo-dGTP and 8oxo-GTP as substrates for DNA polymerization, although with poor efficiency compared to the incorporation of undamaged nucleotides opposite either 8oxodG or undamaged templates. This suggests that *Sp*Pol4 is specialized in tolerating 8oxodG as a DNA template, without contributing significantly to the accumulation of this lesion in the DNA.

## INTRODUCTION

Reactive oxygen species (ROS) arise during cellular aerobic metabolism and through the action of exogenous agents such as ionizing radiation or H_2_O_2_ (1). ROS accumulation can damage diverse cellular components, and is responsible for one of the most common threats to genetic stability: DNA oxidation (2). The most frequent DNA lesion generated by oxidative stress is 7,8-dihydro-8-oxoguanine (8oxoG), reaching steady-state levels of ∼10^3^ to 10^4^ lesions per mammalian cell (3,4). 8oxoG is commonly found in DNA as a deoxyribonucleoside form (8oxodG) that is highly pre-mutagenic, because it does not block DNA synthesis and replicative DNA polymerases can efficiently insert the wrong dA opposite the lesion. Thus, 8oxodG pairs with dC when it adopts the regular *anti* conformation (5), but it can also mispair with dA when 8oxodG adopts a *syn* conformation (6), eventually leading to dG:dC to dT:dA transversion mutations (7,8). The mutagenic potential of 8oxodG together with the association of ROS with carcinogenesis, aging and neurodegenerative diseases make the study of how cells deal with this lesion especially relevant (9,10,11).

Cells have developed multiple mechanisms to reduce ROS toxicity and to eliminate 8oxodG (1), being base excision repair (BER) the principal pathway that repairs this lesion. In *Escherichia coli*, a three-component mechanism, referred to as 8-oxoG (GO) system, prevents 8oxodG mutagenicity (12). It comprises two DNA glycosylases, MutM and MutY, and also an 8oxo-dGTPase named MutT. MutM removes 8oxodG preferentially paired to dC in DNA, but if it fails to repair the damage, 8oxodG can promote dA misincorporation, probably during DNA replication. At this point, MutY excises dA from the 8oxodG:dAMP mispair to provide a second opportunity to restore the original sequence. On the other hand, MutT hydrolyses 8oxo-dGTP and 8oxo-GTP from the oxidized nucleotide pool to prevent their incorporation into DNA. In addition to these three proteins, other enzymes have been suggested to take part in the repair of 8oxodG, such as Nei, which excises 8oxodG from 8oxoGd:dAMP mispairs (13), and Nth which eliminates 8oxodG paired to dG (14). The GO system is highly conserved through evolution from bacteria to higher eukaryotes. Mammalian cells feature homologs of MutM, MutY and MutT named OGG1, MYH and MTH1, respectively (15,16); they also present a *N*th homolog and a paralog of Nei, named hNTH1 and OGG2, respectively (14,17,18). Remarkably, despite the relevance of the GO system in the maintenance of genetic stability, some of these enzymes have not been identified in yeast. The budding yeast *Saccharomyces cerevisiae* displays two MutM homologs, Ogg1 and Ogg2, but lacks MutY and MutT homologs (19). The fission yeast *Schizosaccharomyces pombe* lacks MutM and MutT homologs. Little is known about how 8oxoG is repaired in *S. pombe* but in the absence of a MutM homolog—a crucial enzyme to eliminate 8oxodG from DNA—persistence of this lesion should promote a more frequent misincorporation of dATP. To combat this threat, the MutY homolog glycosylase is conserved in fission yeast (*Sp*MYH) (20).

Over the last decade, specialized DNA polymerases emerged as key players in several DNA repair pathways, including the repair of 8oxodG. Among them, specific DNA polymerases from family X (PolX) are crucial for gap-filling reactions coupled to BER, for the repair of double strand breaks (DSBs) by non-homologous end-joining (NHEJ), and for generating variability during V(D)J recombination (21,22). Four members of the family have been identified in mammals: Polβ, Polμ, Polλ and terminal deoxynucleotidyl transferase (TdT). In addition to its role in BER, NHEJ and V(D)J recombination, Polλ participates in 8oxodG tolerance and repair by virtue of its specific interaction with MYH (23). Thus, after dA removal from the 8oxodG:dA mispair by MYH, Polλ incorporates the correct dCTP opposite 8oxodG with high efficiency and almost exclusively when the reaction is modulated by Replication Protein A (RPA) and Proliferating Cell Nuclear Antigen (PCNA) (24); after tolerating the lesion, Polλ efficiently extends the correct base pair (8oxodG:dCMP) formed (25).

Interestingly, only one family X DNA polymerase is found in yeast, Pol4, which shares biochemical properties with other members of the PolX family such as its 5′-phosphate-mediated affinity for small DNA gaps (a general property of all PolX enzymes), and its intrinsic deoxyribose phosphate (dRP) lyase activity like Polβ and Polλ (26,27). Strikingly, Pol4 stands out among other PolX enzymes due to its capacity to use ribonucleotides (NTPs) and deoxynucleotides (dNTPs) with similar efficiency (26,27), a property that is only mirrored by Polμ and TdT, and that it was proposed to be relevant during NHEJ (28,29,30). Although the function of Pol4 *in vivo* remains unclear, all these properties suggest a role of the polymerase in BER and NHEJ. Recent studies with the *S. pombe* orthologue (*Sp*Pol4) have demonstrated its involvement during DNA gap-filling steps associated to NHEJ (31) or microhomology mediated end-joining (MMEJ) (32).

In this work, we have analysed whether *Sp*Pol4, as Polλ in humans, is involved in the repair and tolerance of 8oxodG lesions, both during gap-filling and NHEJ reactions. Our results suggest that ATP is transiently inserted opposite 8oxodG by *Sp*Pol4, but readily eliminated by RNase H2 and substituted for dCTP. Moreover, analysis of 8oxo-dGTP and 8oxo-GTP incorporation suggests that *Sp*Pol4 may not contribute significantly to their accumulation in the DNA, which is in line with the absence of a MutT homolog in *S. pombe* and will be further discussed.

## MATERIALS AND METHODS

### DNA and proteins

Synthetic DNA oligonucleotides were purchased from Invitrogen and purified by 8 M urea–20% polyacrylamide gel electrophoresis (PAGE). The oligonucleotides used in this work were: Sp1C (5′-GATCACAGTGAGTAC-3′); DG1 (5′-P-AGATACACTTCT-3′); T13N (5′-AGAAGTGTATCTXGTACTCACTGTGATC-3′) were ‘X’ is A, C, G, T or 8oxodG; D1 (CGGAGGGAGGG-3′); D2 (5′-GGGACGTGAGTGCGCG-3′); D3+C (5′-CCCTCCCTCCGCGGCC-3′); D4GX (5′-CGCGCACTCACGTCCCXGGCC-3′) were ‘X’ is G or 8oxodG; Sp1C-N-DG1 (5′-GATCACAGTGAGTACNAGATACACTTCT-3′) where N is dA, dC, C or A.

To build 1 nucleotide (1nt)-gapped molecules, labelled primers Sp1C and was hybridized to templates T13N and downstream DG1. For NHEJ assays, labelled D3+C was used as primer and hybridized to D1 to obtain 3′-protruding substrates. D4GX was used as the cold template and hybridized with D2 to also obtain 3′-protruding molecules. For the ribonucleotide repair assays, Sp1C-N-DG1 molecules were labelled and hybridized to T13X. Oligonucleotides DG1 and D2 contained a 5′-phosphate when indicated.

All the primers were 5′-labelled with [γ-^32^P] ATP and T4 nucleotide kinase for 45 min at 37°C. Hybridizations were performed in buffer 50 mM Tris–HCl pH 7.5 /0.3 M NaCl for 20 min at 65**°**C. Ultrapure unlabelled dNTPs, and NTPs were purchased from GE healthcare. 8oxo-dGTP and 8oxo-GTP were purchased from TriLink Biotechnologies. [γ-^32^P]ATP (3000 Ci/mmol) was obtained from Perkin-Elmer. T4 DNA ligase and T4 polynucleotide kinase were acquired from New England Biolabs. GST-tagged *Sp*Pol4 (GST-*Sp*Pol4) was purified as described in (26).

### Strains and growth conditions

Fission yeast cells were grown and manipulated according to standard protocols in either yeast extract (YES) or minimal medium (EMM) (33). Appropriate amino acids were added to the medium when required to a final concentration of 250 mg/l. The strains involving Pol4 deletion (*Δpol4*) carried the construction pol4::KanMX6 previously described in (26).

To overproduce GST:*Sp*Pol4, the *h^−^ ura4-D18 pDS473(GST)*:Pol4 and *h^−^ ura4-D18 pDS473(GST)* strains were generated by transforming the *h^−^ ura4-D18 Δpol4* strain by the lithium acetate protocol (34) with pDS473(GST) control plasmid or the pDS473(GST):*pol4* plasmid, in which *pol4* is under the control of the thiamine-repressible *nmt* promoter (15 μM of thiamine were added for full repression). These strains were grown exponentially in EMM medium containing thiamine (15 μM). Subsequently, the cells were washed three times and resuspended in the same medium without thiamine. After 18 h of overproduction, 3 × 10^8^ cells were collected to obtain samples for cell extracts.

The *h^−^ ura4-D18 leu1-32 ade6-M26* (provided by Dr A. Pastink (Leiden University Medical Center)) and the *h^+^ ura4-D18 leu1-32 ade6-704 Δrnh201:kanMX* (provided by Prof. Antony Carr (University of Sussex, GDSC)) strains were used to study RNase H2 mediated removal of ribonucleotides paired to 8oxodG. The strains were grown in EMM supplemented with the appropriated amino acids until logarithmic phase (0.6–0.8 O.D._595 nm_) and a sample of 3 × 10^8^ cells was collected.

To induce cell cycle arrest in G1 phase, the thermosensitive strains *cdc10-129* (provided by Dr S. Moreno (Institute of Functional Biology and Genomics, Spain)) and *cdc10–129 Δpol4* were grown in EMM until they reached logarithmic phase (0.6–0.8 O.D._595 nm_). Subsequently, cells were arrested at the restrictive temperature of 36°C during 4 h and samples of each culture were collected to obtain whole cell extracts (WCE). To obtain samples from cells arrested in G2 phase, the thermosensitive *cdc25-22* (provided by Prof. Juan Jiménez (Universidad Pablo de Olavide, Spain)) strain was used following the same protocol described for *cdc10-129* mutants.

To induce arrest in mitosis, the thermosensitive *nda3KM*-*311 leu1-32* ((provided by Dr S. Moreno (Institute of Functional Biology and Genomics, Spain)) strain was grown exponentially in YES medium until it reached logarithmic phase (0.6–0.8 O.D._595 nm_). Cells were subsequently arrested at the restrictive temperature of 19°C for 6 h and a sample from the culture was collected.

### Whole cell extract preparation

Samples of 3 × 10^8^ cells of each culture in asynchronous and arrest conditions were collected and washed with stop buffer (NaCl 150 mM, NaF 50 mM, Ethylene-diamine-tetraacetic acid (EDTA) 10 mM and NaN_3_ 1 mM, pH 8). Pellets were kept at −80°C. The frozen cells were thawed on ice and resuspended in 50 μl of cold lysis buffer (Phosphate buffered saline (PBS) 1X, NaF 50 mM, Ethylene diamine tetraacetic acid (EDTA) 2 mM pH 8, NP-40 1%, *p*-NH_2_-benzamidine 1.3 mM, Phenyl methane sulfonyl fluoride (PMSF) 1 mM and a protease inhibitor cocktail (Roche)). Cells were broken using 750 mg of glass beads (0.4 mm, Sigma) for 15 s (three times) in a Fast-prep machine (Bio101 Inc.) and the crude extract was recovered by 0.2 ml of lysis buffer. After 30 min of centrifugation (15 000 rpm), the soluble fraction was collected and kept at −80°C. Protein concentration was determined by Bicinchoninic acid (BCA) protein assay kit (Thermo Scientific Pierce).

### DNA polymerization assays

For the analysis of the gap-filling activity, the Sp1C/DG1/T13N ^32^P-labelled 1nt-gapped substrates were incubated with purified GST-*Sp*Pol4 (35 nM). The reaction was performed in a total volume of 20 μl containing 1× reaction buffer (50 mM Tris–HCl/ pH 7.5, 1 mM dithiothreitol (DTT), 4% glycerol and 0.1 mg/ml BSA), 2.5 nM of the DNA substrate, the indicated concentration of the NTPs or dNTPs and 2.5 mM MgCl_2_. Reactions were incubated for 15 min at 30**°**C and stopped by adding gel loading buffer (95% (v/v) formamide, 10 mM EDTA, 0.1% (w/v) xylene cyanol and 0.1% (w/v) Bromophenol Blue). Primer extension was analysed by 8 M urea/20% PAGE and autoradiography. When indicated, the percentage of extended primers was quantified by densitometry of the autoradiographs.

For the NHEJ polymerization assays, the D3+C/D1 DNA primer labelled molecule (5 nM) and the D4GX/D2 unlabelled DNA template molecule (12.5 nM) were incubated with purified GST-*Sp*Pol4 (200 nM). The reaction was carried out for 1 h at 30°C in a final volume of 20 μl of reaction buffer that contained variable concentrations of the NTPs and 1 mM MnCl_2_. Trans-directed primer extension was analysed as described above by 8 M urea/20% PAGE and autoradiography. When indicated, the percentage of primers extended with a given nucleotide was quantified by densitometry of the autoradiographs.

To study gap-filling activity present in cell extracts, the Sp1C/DG1/T13N 1nt-gapped molecule (2.5 nM) was incubated with 20 μg of the WCE. The reaction mixture, in 20 μl, was as described above, but containing 1 mM MnCl_2_ and variable concentrations of NTPs or dNTPs. Reactions were incubated for 20 min at 30°C, and primer extension was analysed as described above by 8 M urea/20% PAGE and autoradiography.

For the NHEJ reactions with cell extracts, we incubated 30 μg of WCE with reaction mixture (in 20 μl), the D3+C/D1 labelled (5 nM) and the D4GX/D2 cold (12.5 nM) DNA molecules, the indicated NTPs and 1 mM MnCl_2_. Reactions were performed during 30 min at 30°C, and primer extension was analysed as described above by 8 M urea/20% PAGE and autoradiography.

### Steady-state kinetics assays

Kinetic parameters of 1nt gap-filling reactions mediated by *Sp*Pol4 were analysed as described previously (35). The reaction mixture (20 μl) contained 50 mM Tris–HCl pH 7.5, 1 mM DTT, 4% glycerol, 0.1 mg/ml BSA, 0.2 μM of the gapped DNA substrate and 2–8 nM of purified *Sp*Pol4. Reactions were initiated by adding the nucleotide at different concentrations ranging from 0.01 to 25 μM, except for dCTP incorporation opposite 8oxodG and 8oxo-GTP incorporation opposite dA where the concentrations used ranged from 0.1 to 250 μM and dGTP incorporation opposite dC where the concentrations used ranged from 0.001 to 2.5 μM. After incubation for 5 min at 30°C, reactions were stop by adding loading buffer. Primer extension was analysed by 8 M urea/20% PAGE and gel band intensities were quantified using a BAS reader 1500 (Fujifilm). The observed rate of nucleotide incorporation (extended primer) was plotted as a function of nucleotide concentration and the data were fit to the Michaelis–Menten equation using non-linear regression to determine the apparent *K*_m_ and *V*_max_ parameters. The relative insertion efficiency (*f***°**ins) was determine using the following equation *f***°**ins = (*k*_cat_/*K*_m_)_damaged_/(*k*_cat_/*K*_m_)_undamaged_, where *k*_cat_ = *V*_max_/[enzyme]_total_.

### Ribonucleotide excision assays

To analyse RNase H2 activity present in WCE, the indicated Sp1C-N-DG1/T13X dsDNA (2.5 nM) was incubated with 30 μg of the WCE in 20 μl of reaction mixture containing 50 mM Tris–HCl/pH 7.5, 2.5 mM MgCl_2_, 1 mM DTT, 4% glycerol and 0.1 mg/ml BSA) for the indicated times at 30°C. DNA incision by RNase H2, was analysed by 8 M urea/20% PAGE and the labelled 15-mer product was quantified by densitometry of the autoradiographs.

To study the subsequent polymerization after RNase H2 incision, 30 μg of the WCE were incubated with the Sp1C-A-DG1/T138oxoG labelled dsDNA molecule (2.5 nM) in 20 μl of reaction mixture described above, with the addition of the indicated amounts of NTPs and/or dNTPs, during 30 min at 30°C.

### Western blot

Whole cell extracts were separated by using 10% SDS-polyacrylamide gels and the proteins were transferred to nitrocellulose membranes. Overproduced GST-*Sp*Pol4 and α-tubulin were detected using anti-GST (G1160, Sigma) and anti-tubulin (T6074, Sigma) monoclonal antibodies, respectively. Enhanced chemiluminescence detection (Amersham Biosciences) and anti-mouse IgGs coupled to horseradish peroxidase antibody (Amersham Biosciences) were used for protein visualization.

## RESULTS

### *Sp*Pol4 tolerates 8oxodG, preferably in an error-prone manner

**Figure 1.**
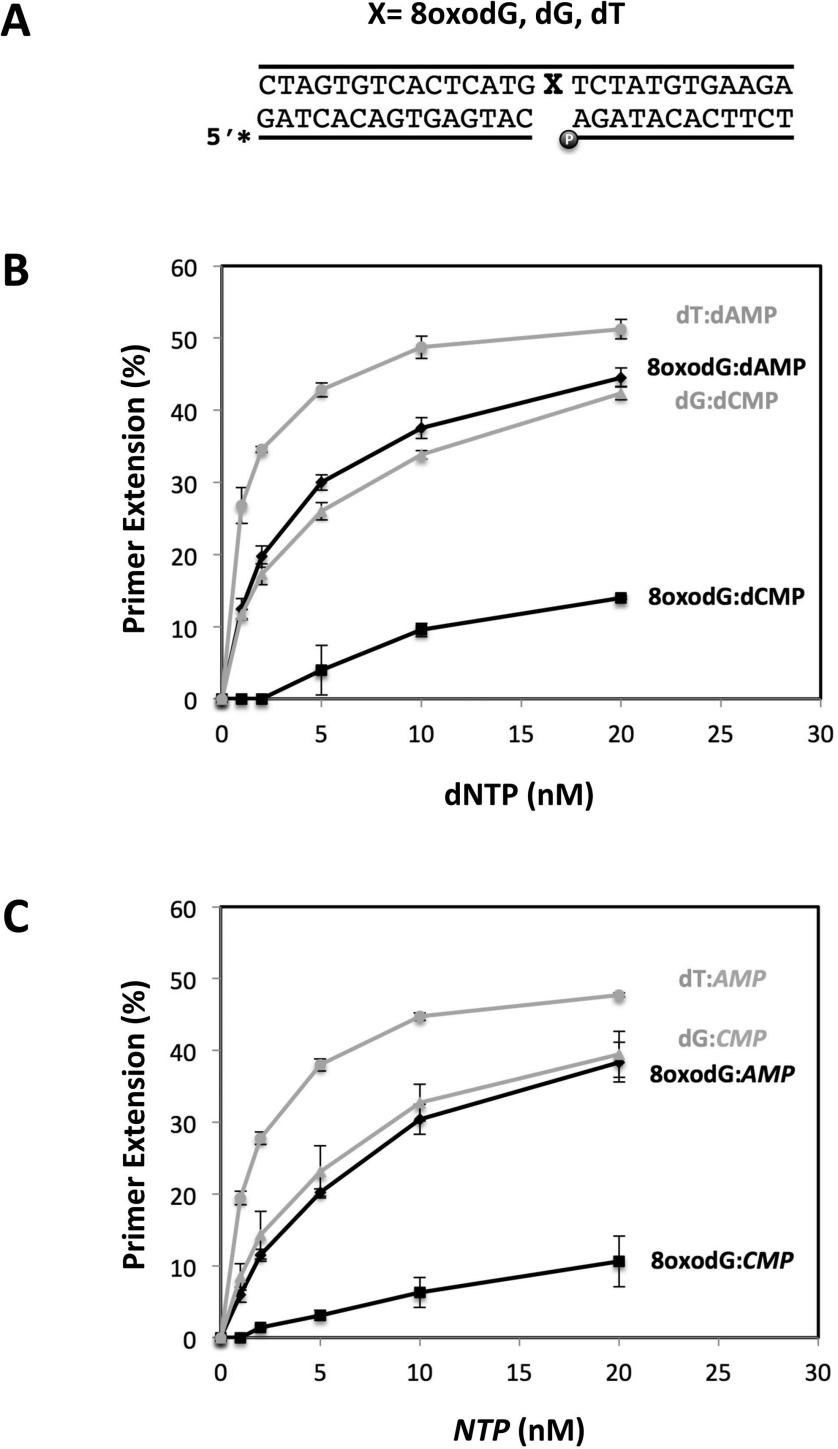
Error-free versus error-prone incorporation opposite 8oxodG by *Sp*Pol4 *in vitro*. (**A**) Scheme of the labelled substrates used to determine the gap-filling activity of *Sp*Pol4 *in vitro*. The molecules contained a 1nt-gap (X) with 8oxodG, dG or dT in the gap position and a recessive 5′-phosphate flanking the gap. (**B**) Primer extension (gap-filling) by purified GST-*Sp*Pol4 (35 nM), templated either by 8oxodG, dT or dG, with the indicated amounts of dCTP or dATP (*n* = 3). After incubation at 30°C for 15 min, samples were processed as described in the ‘Materials and Methods’ section. (**C**) The same gap-filling analysis performed in (B) but using the indicated amounts of either ATP or CTP (*n* = 3).

To elucidate a possible role of *Sp*Pol4 in the tolerance of 8oxodG, we first evaluated its efficiency and preference of incorporation of dNTPs opposite the lesion. For this, purified *Sp*Pol4 was assayed on a 1nt­-gapped DNA molecule either with dG, 8oxodG, or dT in the gap position (X in Figure 1A). This DNA molecule, that mimics BER and NHEJ intermediates, has a recessive 5′-phosphate flanking the gap, which increases the efficiency of DNA polymerization by purified *Sp*Pol4 (26). As shown in Figure 1B, *Sp*Pol4 could incorporate dCTP and dATP opposite 8oxodG but, strikingly, it performed the error-prone reaction (8oxodG:dAMP) with much higher efficiency. Moreover, incorporation of dATP opposite 8oxodG was only around 3-fold less efficient than opposite dT, and similar to the insertion of dCTP opposite dG. Likewise other PolXs, such as Polμ and TdT, *Sp*Pol4 has the extraordinary ability to incorporate NTPs to a DNA primer (26). Remarkably, *Sp*Pol4 could tolerate 8oxodG using NTPs (Figure 1C), and as efficiently as using dNTPs. Again, *Sp*Pol4 displayed a similar preference for the error-prone reaction (8oxodG:AMP), that also reached the efficiency of CTP incorporation opposite an undamaged dG (dG:CMP; Figure 1C).

To further confirm these conclusions, we performed kinetic analysis of the gap-filling reactions opposite 8oxodG under steady-state conditions. As shown in Table 1, the catalytic efficiency of dATP insertion opposite 8oxodG was 29-fold higher than dCTP, as *Sp*Pol4 displayed much lower affinity (higher *K*_m_) for this nucleotide, and as efficient as dCTP insertion opposite an undamaged dG. As expected, the catalytic efficiency of ATP insertion was very similar to dATP (only 1.6-fold lower) and 18-fold higher than dCTP insertion opposite the lesion. All together, these results demonstrated that *Sp*Pol4 preferably tolerates 8oxodG in an error-prone manner using either dNTPs or NTPs, and that this lesion does not impair *Sp*Pol4 gap-filling efficiency compared to an undamaged template dG.

**Table 1.**
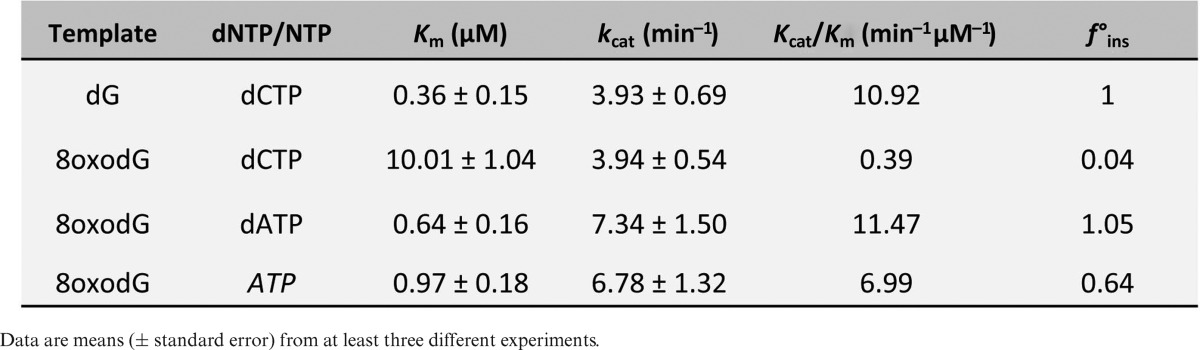
Steady-state kinetic parameters of insertion opposite dG and 8oxodG by purified S*p*Pol4

### *Sp*Pol4 tolerates 8oxodG by using physiological concentrations of ATP

**Figure 2.**
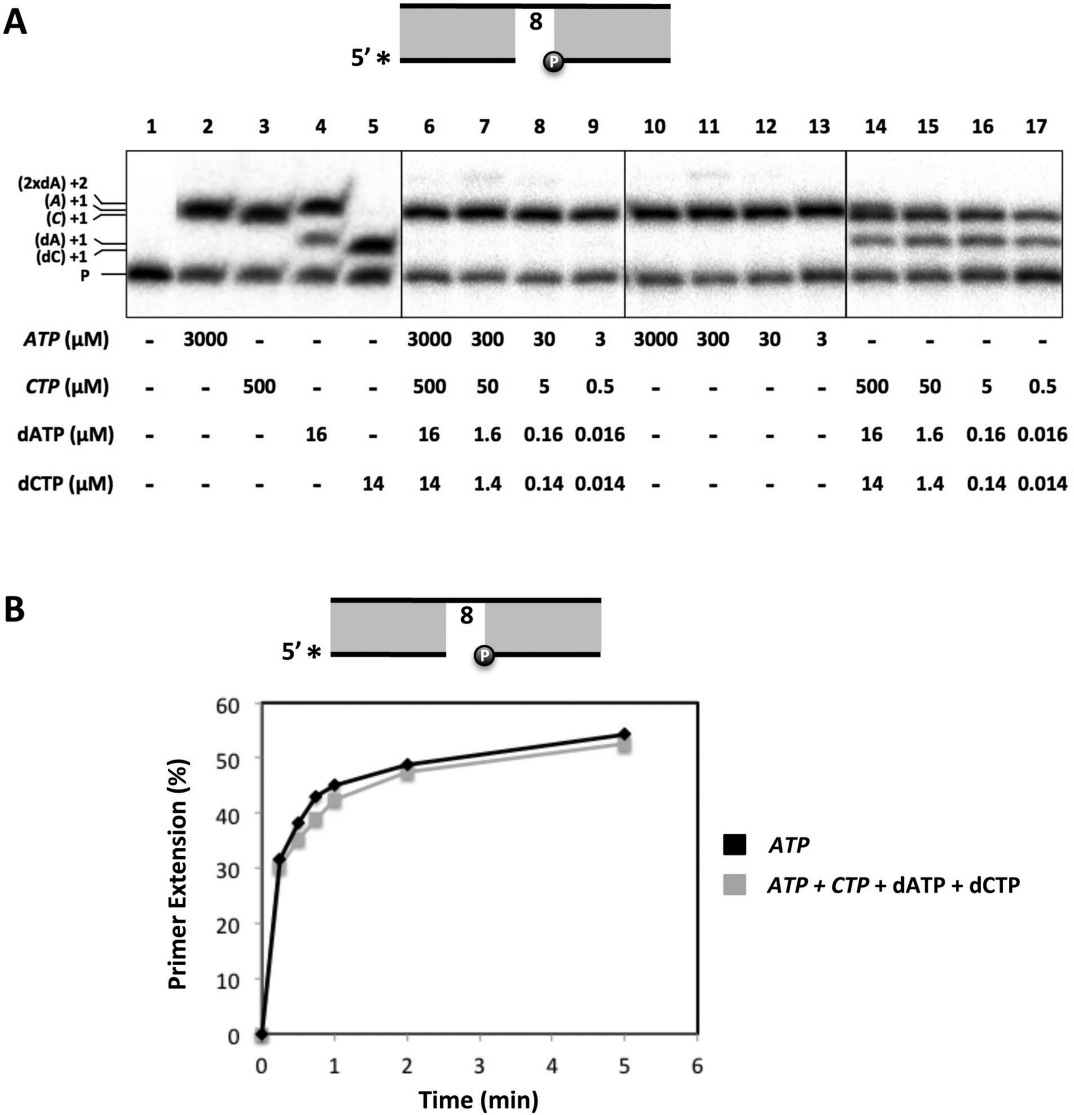
*In vitro* tolerance of 8oxodG by *Sp*Pol4 using physiological concentrations of nucleotides. (**A**) Purified GST-*Sp*Pol4 (35 nM) was incubated with the 8oxodG-gapped substrate (2.5 nM), and the indicated combination and concentration of ribo and deoxynucleotides. After incubation at 30**°**C for 15 min, primer extension was analysed as described in the the ‘Materials and Methods’ section. (**B**) Kinetics of ATP incorporation opposite 8oxodG by purified GST-*Sp*Pol4 (35 nM), using a physiological concentration of ATP (3000 μM) either in alone or in the presence of CTP (500 μM), dATP (16 μM) and dCTP (14 μM). After incubation at 30°C for the indicated times, samples were processed as described in the ‘Materials and Methods’ section.

Cellular concentrations of NTPs significantly exceed those of dNTPs, being the levels of ATP particularly elevated. In both budding yeast and human cells, concentration of nucleotide pools is very similar (36,37), but in *S. pombe* it has not been precisely determined, although some studies have estimated dNTP concentrations as a percentage of either total NTP concentration (38) or ATP concentration (39). Remarkably, these studies demonstrate that, likewise in *S. cerevisiae*, dATP and dCTP concentrations are very similar in *S. pombe*, and represent around only 0.4% of the total ATP concentration (39). Thus, 8oxodG tolerance by *Sp*Pol4 was evaluated using the gapped DNA molecule previously used (Figure 1) and the nucleotide concentrations described for *S. cerevisiae in vivo*. Competitive incorporation of NTPs versus dNTPs could be evaluated since they have different molecular weights, and the different +1 extended primers can be separated by gel electrophoresis, as shown in Figure 2A (first panel). Therefore, when a mix of ATP, CTP, dATP and dCTP was provided at their putative physiological concentrations (3 mM ATP; 0.5 mM CTP; 16 μM dATP; 14 μM dCTP), or at lower but proportional concentrations, *Sp*Pol4 generated only one product (Figure 2A, second panel) whose electrophoretic mobility was compatible with the incorporation of a ribonucleotide, perhaps ATP, according to the specific mobility of each independent +1 product (see Figure 2A, first panel). In fact, when ATP was provided alone, *Sp*Pol4 generated the same polymerization product, even with an ATP concentration as low as 3 μM (Figure 2A, third panel). Conversely, when ATP was removed from the nucleotide mix, *Sp*Pol4 generated three distinct products (Figure 2A, fourth panel), two of them corresponding to the incorporation of CTP and dATP, and a minor band likely corresponding to the incorporation of two dATP units (see also Figure 2A first panel) occurring through strand-displacement, due to the presence of a dT templating base contiguous to the 8oxodG lesion (Supplementary Figure S1); interestingly, no band corresponding to incorporation of dCTP was observed, perhaps suggesting a strong and direct competition with CTP. In the presence of all the nucleotides (ATP, CTP, dATP and dCTP) at the physiological concentrations, only ATP appeared to be incorporated, suggesting that all the alternative nucleotides were outcompeted (see also Supplementary Figure S2).

We next evaluated the kinetics of ATP incorporation opposite 8oxodG, at the physiological concentration of 3 mM. The gap-filling reaction performed by *Sp*Pol4 was very efficient and became saturated after 1 min of incubation (Figure 2B), although ∼50% of the primers could not be extended, possibly due to incomplete hybridization. As expected, the kinetics of primer extension with ATP was not affected by the addition of physiological concentrations of CTP, dATP and dCTP (Figure 2B). All together, these results demonstrated that *Sp*Pol4 tolerates 8oxodG by incorporating ATP almost exclusively.

### Error-prone incorporation of ATP opposite 8oxodG is *Sp*Pol4-specific in *S. pombe* cell extracts

**Figure 3.**
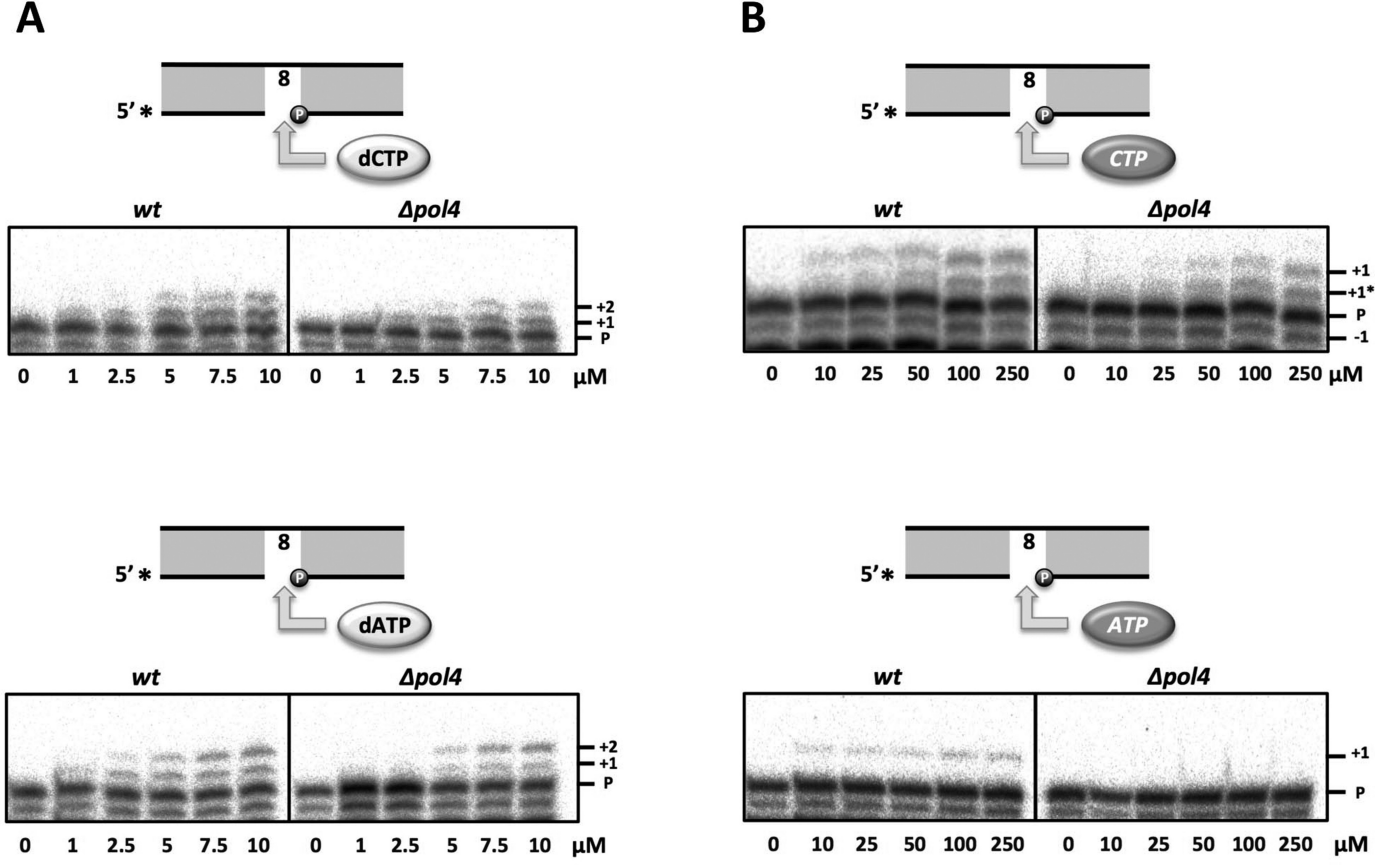
Incorporation of nucleotides opposite 8oxodG by *S. pombe* cell extracts. (**A**) Gap-filling activity by WCE derived from either wild-type or *Δpol4 S. pombe* synchronized in G1. 20 μg of each extract were incubated with the 8oxodG-gapped substrate (2.5 nM), and the indicated amounts of either dCTP (upper panel) or dATP (lower panel). (**B**) Gap-filling experiments were performed as in (A) but using the indicated amounts of either CTP or ATP. After incubation at 30°C for 20 min, primer extension was analysed as described in the ‘Materials and Methods’ section.

To further evaluate the contribution of *Sp*Pol4 to 8oxodG tolerance *in vivo*, we tested the gap-filling activity of *S. pombe* WCE either containing or lacking *Sp*Pol4, and using the same 8oxodG-containing gap described above. It has been demonstrated that NHEJ is the predominant DSB repair mechanism during G1 phase in fission yeast (40); thus, given the possible implication of *Sp*Pol4 in this pathway (26,31), we used G1-synchronized wild-type or *Sp*Pol4-defective (*Δpol4*) extracts. Extracts from each strain could incorporate dCTP opposite 8oxodG (Figure 3A, top), and a second dCTP incorporation mediated by primer slippage (see also Supplementary Figure S1); however, *Sp*Pol4 contribution was not scored at any concentration (Figure 3A, top; compare *wt* and *Δpol4* panels). The wild-type WCE also catalysed the incorporation of dATP opposite 8oxodG (Figure 3A, bottom), and a subsequent dATP incorporation mediated by strand-displacement (see also Supplementary Figure S1); however, *Δpol4* WCE failed to promote misinsertion of dATP opposite 8oxodG at low concentrations (1–2.5 μM), supporting an *Sp*Pol4 contribution (Figure 3A, bottom).

Incorporation of CTP by both extracts also produced two different bands (Figure 3B, top): the slower migrating band (+1) corresponded to a direct insertion opposite 8oxodG, and the faster migrating band (+1*), which corresponds to its insertion opposite an undamaged dG (preceding the 8oxodG lesion) occurring onto partially degraded (−1) primer molecules (see also Supplementary Figure S1). Similarly to dATP, CTP incorporation opposite 8oxodG (+1 product) was not detected with *Δpol4* WCE when using low CTP concentrations (10–25 μM; Figure 3B, top), supporting an *Sp*Pol4 contribution when reading the 8oxodG lesion; conversely, and mimicking the situation with dCTP, we could not detect an *Sp*Pol4 contribution to the +1* product (inserted opposite an undamaged dG templating base) at any concentration. Remarkably, incorporation of ATP opposite 8oxodG produced a single product, which was completely *Sp*Pol4-specific at any concentration tested, as it was undetectable in the WCE lacking *Sp*Pol4 (Figure 3B, bottom).

These results suggest that *in vivo*, other polymerases in addition to *Sp*Pol4 may tolerate 8oxodG by incorporating dATP/dCTP and even CTP. However, *Sp*Pol4 appears to be quite efficient and specific to read the 8oxodG lesion with ATP, an error-prone outcome favouring transversion mutations.

### *Sp*Pol4 inserts ATP opposite 8oxodG during NHEJ

**Figure 4.**
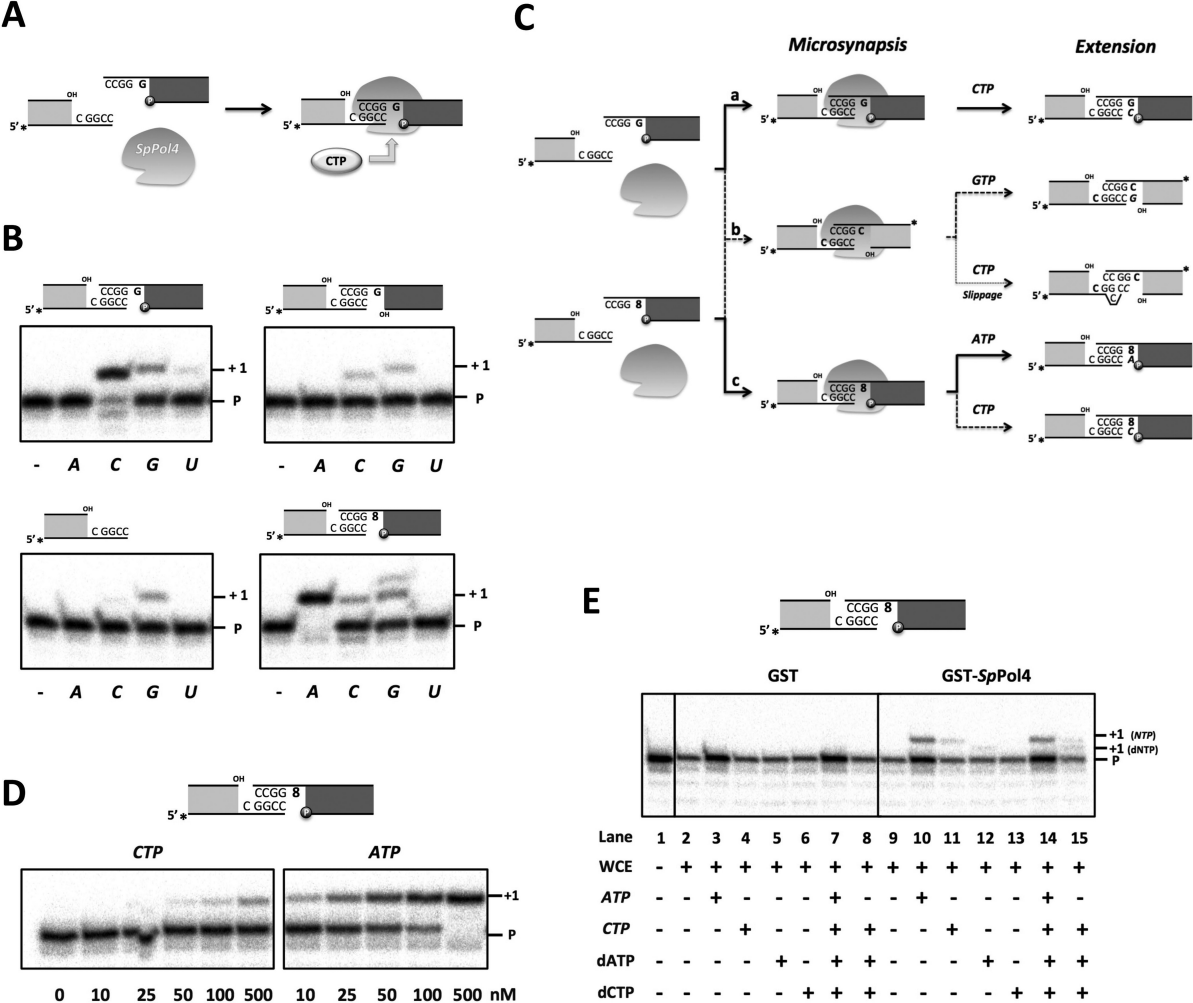
NHEJ coupled to 8oxodG tolerance by *Sp*Pol4. (**A**) Scheme of *Sp*Pol4 bridging two 3′-protruding DNA molecules used for NHEJ experiments. (**B**) NHEJ by GST-*Sp*Pol4 (200 nM) using a labelled 3′-protruding primer molecule (5 nM) either alone or with a set of different 3′-protruding cold template molecules (12.5 nM). ATP, CTP, GTP or UTP (500 nM) were added to the reaction when indicated. After incubation at 30°C for 60 min, NHEJ was analysed as described in the ‘Materials and Methods’ section. (**C**) Schematic representation of the different outcomes of the NHEJ experiments shown in (B). (**D**) Nucleotide insertion opposite 8oxodG during NHEJ, analysed as in (B), but using the indicated amounts of either CTP or ATP. The relative efficiency of incorporation of ATP versus CTP opposite 8oxodG (20-fold higher) was quantified as described in the ‘Materials and Methods’ section. (**E**) Tolerance of 8oxodG during NHEJ using asynchronous WCE overexpressing either GST or GST-*Sp*Pol4 (30 μg) and physiological concentrations of ATP (3000 μM), CTP (500 μM), dATP (16 μM) and dCTP (14 μM), added as indicated. After incubation at 30°C for 30 min, NHEJ was analysed as described in the ‘Materials and Methods’ section. Ribonucleotides are denoted in italics.

DSBs are frequently associated with other kinds of lesions such as abasic sites, dRP residues and also damaged bases like 8oxodG (41). Thus, damage tolerance associated to DSB repair can be essential to overcome this dangerous form of DNA damage. Firstly, we wanted to know whether *bona fide* NHEJ could be performed *in vitro* by *Sp*Pol4 with NTPs, and in the absence of core factors, as recently shown for human Polμ (30). For that we used two different dsDNA molecules with 3′-protruding, partially complementary ends. After microsynapsis of the two ends, if bridged by the NHEJ polymerase, these molecules would form two 1nt gaps, one of which contains a templating dG adjacent to a connection of 4 bp of complementarity (Figure 4A). Based on previous work with human Polμ and Polλ (42), we can anticipate that a recessive 5′-phosphate exclusively present in the unlabelled DNA molecule, flanking the dG-containing gap, would orient *Sp*Pol4 to extend the labelled primer with CTP.

In these conditions, we observed an efficient +1 extension of the labelled primer only when CTP was provided, as expected from a *bona fide* NHEJ reaction (Figure 4B, top left, and Figure 4Ca). Under the same reaction conditions, when dCTP was added at similar concentrations, the efficiency of NHEJ was comparable (Supplementary Figure S3A). Thus, the nearly complete lack of sugar discrimination described for *Sp*Pol4 during gap-filling (26) is also valid during NHEJ.

As anticipated, this preferred insertion of CTP was drastically reduced if a 5′P was not present at the unlabelled (template) DNA end (Figure 4B, top right). As a control to evaluate the existence of some terminal transferase activity with NTPs, that could explain the minor extension obtained with GTP and UTP, only the labelled end (acting as primer) was provided in the presence of each NTP at 0.5 μM. In this case, *Sp*Pol4 did not incorporate ATP or UTP, but catalysed some GTP and CTP incorporation (Figure 4B, bottom left). Although some terminal transferase activity cannot be formally discarded, we favour that the incorporation of GTP could have occurred as a NHEJ event in which two labelled molecules form two 1nt gaps with a dC directing the insertion (Figure 4Cb). On the other hand, the lower background with CTP observed by providing only the primer supports the *bona fide* NHEJ reaction shown in Figure 4B (top left), but it could be also due to a NHEJ event involving two identical (labelled) ends and misalignment of the connected primer (Figure 4Cb).

To evaluate whether *Sp*Pol4 could tolerate 8oxodG during NHEJ, we performed the same experiment, but using a template molecule bearing an 8oxodG in the gap position. Under these conditions, *Sp*Pol4 preferentially incorporated ATP, and much less CTP and GTP (Figure 4B, bottom right, and Figure 4Cc). The lack of any background with ATP in the previous experiments supports a *bona fide* NHEJ reaction where 8oxodG is preferentially recognized by *Sp*Pol4 in the *syn* conformation, directing insertion of ATP. It is also worth noting that most of the incorporated CTP in this experiment is likely templated by 8oxodG, by comparison with the experiment shown in Figure 4B (bottom left). Further analysis at different nucleotide concentrations demonstrated that *Sp*Pol4 tolerates 8oxodG during NHEJ by incorporating ATP with ∼20-fold higher efficiency than CTP (Figure 4D). On the other hand, GTP incorporation was similar to the background detected in all the previous experiments, which again suggests that it is occurring through the synapsis of two labelled primer molecules (Figure 4Cb). The very same conclusions can be obtained when the incorporation of dATP and dCTP opposite 8oxodG was tested during NHEJ: dATP was favoured over dCTP (Supplementary Figure S3B), but it can be predicted that ATP would be the preferred nucleotide inserted opposite 8oxodG given the physiological concentration of each of these substrates.

Endogenous levels of *Sp*Pol4 were insufficient to study NHEJ reactions using WCE (data not shown). Thus, to gain further insights on the 8oxodG tolerance reactions carried out by *Sp*Pol4 during NHEJ in the presence of other core factors, we obtained asynchronous WCE derived from cells overexpressing GST-tagged *Sp*Pol4 (Supplementary Figure S4). These extracts were incubated with the same 3′-protruding DNA molecules described before, and physiological concentrations of ATP, CTP, dATP or dCTP, either alone or in combination. These WCE tolerated 8oxodG during NHEJ using ATP, CTP or dATP, but only if GST-*Sp*Pol4 was over-expressed (Figure 4E, lanes 10–12). In agreement with our *in vitro* data, *Sp*Pol4 preferably incorporated ATP during the repair process (Figure 4E, lane 10), when other NHEJ repair factors provided by the extract were also present. Moreover, *Sp*Pol4 incorporated ATP almost exclusively in the presence of physiological concentrations of CTP, dATP and dCTP (Figure 4E, compare lanes 14 and 15).

All together, these data indicate that *Sp*Pol4 (either in the absence or presence of NHEJ core factors) can perform *bona fide* NHEJ and tolerate 8oxodG preferentially with ATP.

### RNase H2 present in *S. pombe* cell extracts efficiently targets 8oxodG:AMP mispairs

**Figure 5.**
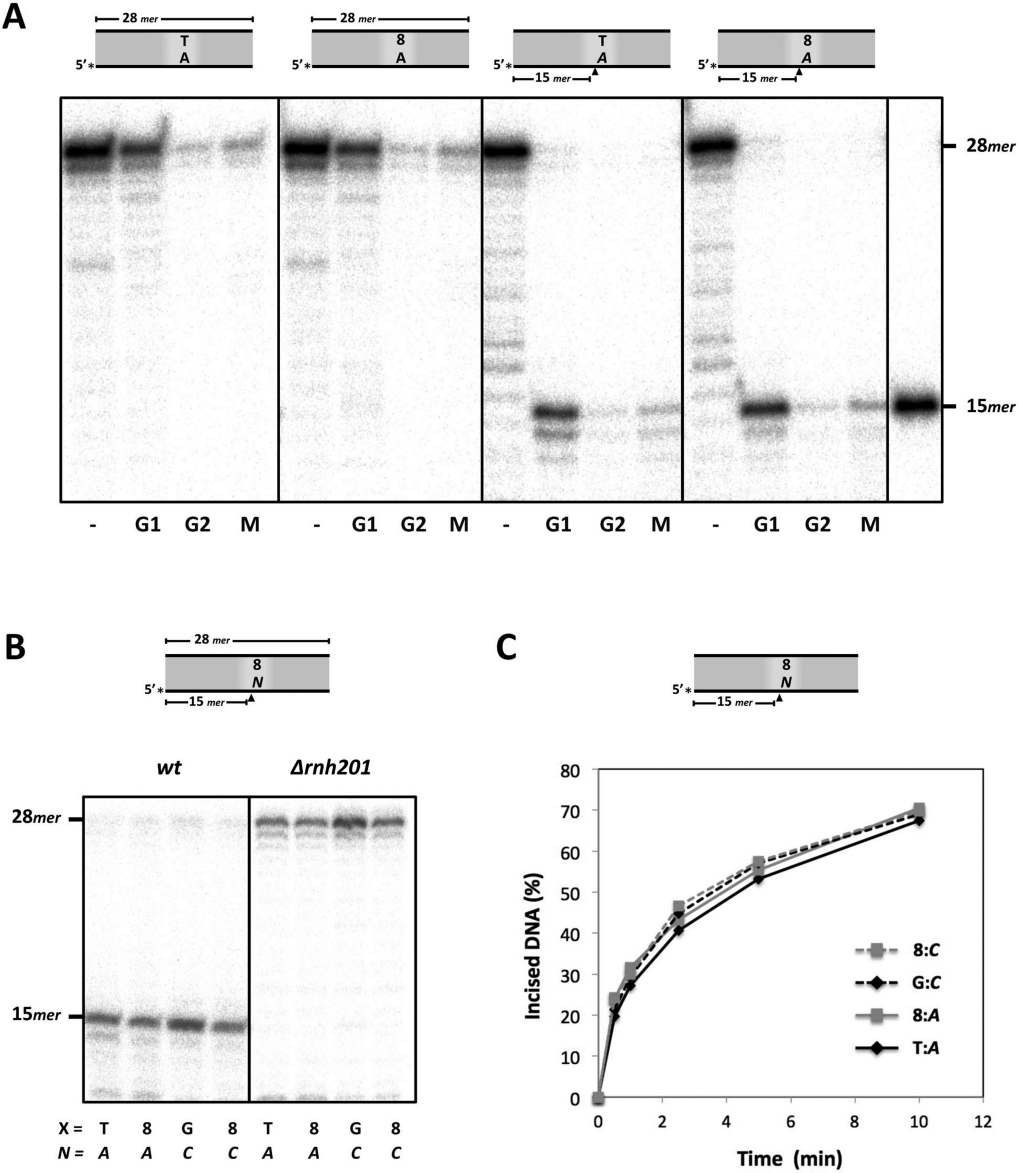
RNase H2-mediated 5′-incision of ribonucleotides paired to 8oxodG. (**A**) Ribonucleotide incision experiments performed with labelled dsDNA molecules (2.5 nM; 28*mer*) containing dT:dAMP, 8oxodG:dAMP,dT:AMP or 8oxodG:AMP bases pairs that were incubated separately with WCE synchronized in G1, G2 or M (30 μg). These molecules were also incubated without WCE (−), to be used as a marker of the intact substrate. A labelled oligonucleotide (Sp1C; 15*mer*) was used as an appropriate molecular marker. After 30 min of incubation at 30°C, incision of the DNA was analysed as described in the ‘Materials and Methods’ section. (**B**) Wild-type and RNH201 null (*Δrnh201*) asynchronous WCE were incubated with labelled dsDNA molecules (2.5 nM) containing either dT:AMP, 8oxodG:AMP, dG:CMP or 8oxodG:CMP base pairs embedded, and processed as in (A). (**C**) Kinetics of ribonucleotide incision by RNase H2 present in wild-type G1 WCE (30 μg), performed as in (A). The percentage of 15*mer* product generated by RNase H2 is designated as incised DNA (%). Ribonucleotides are denoted in italics.

The high cellular concentrations of NTPs make these nucleotides valuable substrates for DNA repair and tolerance reactions; however, their persistence in the genome is undesirable as it renders DNA more susceptible to hydrolysis. In eukaryotic organisms, RNase H2 initiates the removal of single NMPs embedded within a DNA sequence (43); however, whether RNase H2 or other enzymes can initiate ribonucleotide repair of 8oxodG:AMP base pairs has not been reported yet. To evaluate this hypothesis, labelled dsDNA molecules containing a single dT:dAMP, dT:AMP, 8oxodG:dAMP or 8oxodG:AMP base pair in a central position were incubated with *S. pombe* cell extracts, corresponding to synchronized G1, G2 or M cell-cycle phases. None of the extracts incised the dsDNA at the central position corresponding to either dT:dAMP or 8oxodG:dAMP (Figure 5A; first and second panels), despite a reported *Sp*MYH-like activity that could eliminate dA paired to 8oxodG (20). Conversely, the extracts efficiently incised the labelled strand, at the 5′ side of the ribo-adenosine (*A* in the figure), similarly in both DNA substrates containing either dT:AMP or 8oxodG:AMP base pairs (Figure 5A, third and fourth panels). Remarkably, the 15-mer incised product was better detectable in the G1 extract than in both G2 and M extracts, possibly due to higher unspecific nuclease activity in the latter.

Those incised products on DNA molecules containing either dT:AMP or 8oxodG:AMP could be also obtained when using asynchronous wild-type cell extracts, and also when the DNA molecules contained either dG:CMP or 8oxodG:CMP (Figure 5B). RNase H2 is a heterotrimeric enzyme composed of two regulatory subunits (RNase H2 B and RNase H2 C) and a catalytic subunit (RNase H2 A), the latter named RNH201 in yeast (43). When using RNH201 deficient (*Δrnh201)* asynchronous extracts, no incision was detected in all cases (Figure 5B), demonstrating that RNase H2 targets not only single ribonucleotides present in dsDNA, but also those paired to the 8oxodG lesion. Quantitative analysis of the incision kinetics demonstrated that RNase H2 can remove the ribonucleotides paired to 8oxodG as efficiently as when paired to an undamaged base (Figure 5C).

### Elimination of AMP mispaired to 8oxodG triggers specific error-free bypass in *S. pombe* cell extracts

**Figure 6.**
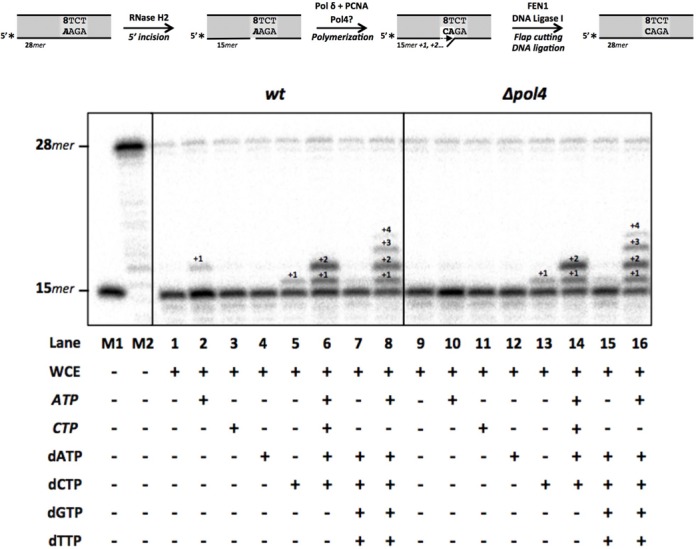
Ribonucleotide excision repair and error-free bypass of 8oxodG using *S. pombe* cell extracts. The top scheme shows the DNA substrate used, and the order of events compatible with the ribonucleotide excision repair (RER) pathway. Wild-type and *Δp*o*l4* WCE synchronized in G1 (30 μg) were incubated with the labelled dsDNA molecule containing the 8oxodG:AMP base and. ATP (3000 μM), CTP (500 μM), dATP (16 μM), dCTP (14 μM), dGTP (12 μM) or dTTP (30 μM) were added to the reaction when indicated. After 30 min at 30°C, incision and further extension was analysed as described in the ‘Materials and Methods’ section. M1, labelled oligonucleotide (Sp1C; 15*mer*) used as a molecular marker. M2, labelled dsDNA molecule (28*mer*) used as substrate for RER. In the figure, ribonucleotides are denoted in italics.

Ribonucleotide excision repair (RER), reconstituted *in vitro* with enzymes purified from *S. cerevisiae*, requires several steps: RNase H2 incision (5′ of the ribonucleotide), strand displacement-mediated DNA polymerization by Pol∂ and PCNA, and finally flap excision and DNA ligation performed by FEN1 and DNA ligase I, respectively (44). In an attempt to reproduce the steps of RER that could be excising ribonucleotides inserted opposite 8oxodG lesions, and could require *Sp*Pol4 activity, we used the dsDNA molecule containing an 8oxodG:AMP base pair (Figure 6; see scheme), physiological concentrations of selected nucleotides, and either wild-type or *Δpol4* WCE (synchronized in G1). Following the activity of RNase H2, some ATP could be incorporated by the wild-type extract but barely by the *pol4-*deficient (Figure 6, lanes 2 versus 10), demonstrating again that although this reaction is limited, it is *Sp*Pol4-dependent; however, if ATP is reinserted during RER, it would be unproductive, as it would restore the excised ribonucleotide. Neither CTP nor dATP (two valid *Sp*Pol4 substrates to be inserted opposite 8oxodG, as shown *in vitro*) were incorporated by the extracts (Figure 6, lanes 3–4 and 11–12). Remarkably, dCTP was inserted as efficiently as ATP opposite 8oxodG, but irrespective of the presence of *Sp*Pol4 (Figure 6, lanes 5 and 13), demonstrating that the excised ribonucleotide can be substituted by dC, thus triggering error-free tolerance of this lesion.

On the other hand, when ATP, CTP, dATP and dCTP were provided together, two polymerization products were generated by both WCE (Figure 6, lanes 6 and 14). The mobility of the upper band (+2) is not compatible either with an ATP incorporation after a dC first insertion, or with a first ATP incorporation, given its intensity and its *Sp*Pol4-independence; therefore, the +2 product likely corresponds to the consecutive insertion of dC and dA, the latter templated by the dT base following the 8oxodG lesion.

Remarkably, no elongation products were observed in the presence of the 4 dNTPs (Figure 6, lanes 7 and 15). However, +1, +2, +3 and +4 products were observed when the four dNTPs were supplemented with ATP (Figure 6, lanes 8 and 16), in agreement with its need for PCNA loading, and further strand-displacement during RER process (44).

Taken together these data demonstrated that AMP paired to 8oxodG is efficiently removed and substituted by dCMP (error-free tolerance), in a process similar to regular RER.

### 8oxo-dGTP and 8oxo-GTP are inefficient substrates during *in vitro* polymerization by SpPol4

**Figure 7.**
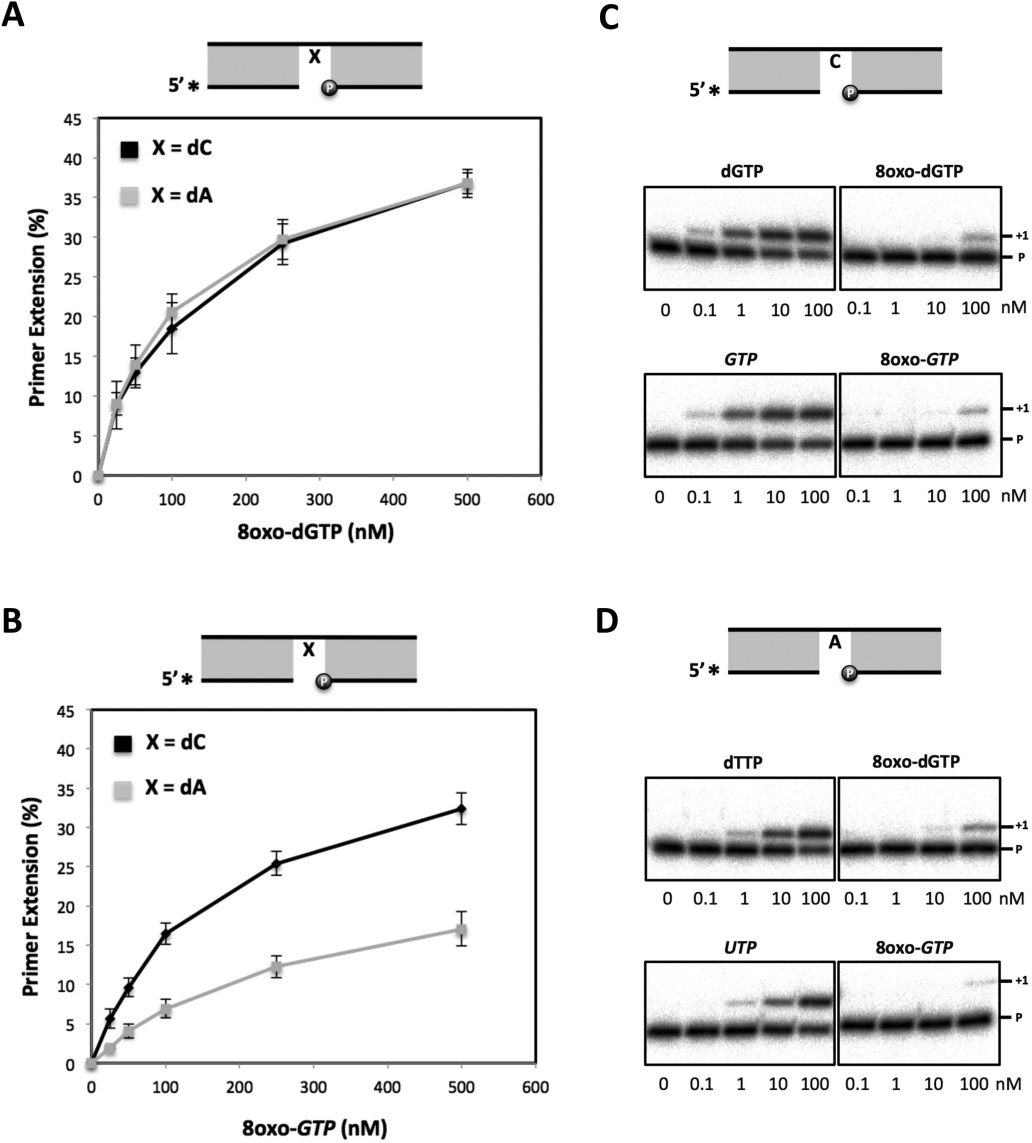
8oxo-dGTP and 8oxo-GTP incorporation by *Sp*Pol4 *in vitro*. (**A**) Primer extension (gap-filling) by purified GST-*Sp*Pol4 (35 nM) opposite templates dC or dA using the indicated concentrations of 8oxo-dGTP (*n* = 3). After incubation at 30**°**C for 15 min, primer extension was analysed as described in the ‘Materials and Methods’ section. (**B**) The same gap-filling experiment described in (A) was carried out using 8oxo-GTP (*n* = 3). (**C**) Comparative incorporation of 8oxo-dGTP/8oxo-GTP versus undamaged dGTP/GTP by purified GST-*Sp*Pol4 (35 nM) opposite the dC-gapped molecule, performed as in (A). (**D**) As in (C) but using dTTP and UTP as undamaged nucleotides and template dA.

ROS can oxidize the cellular pools of nucleotides generating 8oxo-dGTP and 8oxo-GTP, which may abound in *S. pombe* due to the absence of a MutT homolog and could be wrongly incorporated opposite dA. In fact, as 8oxo-dGTP is in *syn* configuration in solution (45), it is ready to be misrecognized (as dTTP) for most DNA polymerases. Given the flexibility of *Sp*Pol4 active site, which allows both ribo and deoxynucleotide substrates and efficiently tolerates 8oxodG template lesions, we evaluated whether *Sp*Pol4 could use 8oxo-dGTP/8oxo-GTP as nucleotides for DNA polymerization. For this, purified *Sp*Pol4 was incubated with a 5′ labelled 1nt-gapped DNA molecule with either dC or dA in the gap position and variable concentrations of either 8oxo-dGTP or 8oxo-GTP. Interestingly, *Sp*Pol4 incorporated 8oxo-dGTP with similar efficiency opposite templates dC or dA (Figure 7A), and 8oxo-GTP opposite template dC better than opposite dA, but similarly to the incorporation of 8oxo-dGTP (Figure 7B). Thus, it is tempting to speculate that the 2′OH group of the ribose is limiting polymerization specifically in the *syn* conformation. To better understand the incorporation of 8oxo-dGTP and 8oxo-GTP by *Sp*Pol4 during gap-filling reactions, and obtain quantitative data, we evaluated the kinetics of their insertion under steady-state conditions. This analysis demonstrated that the catalytic efficiency of 8oxo-dGTP insertion was very similar opposite either template dA or dC (Table 2). Moreover, the catalytic efficiency of 8oxo-GTP incorporation opposite dC was similar to 8oxo-dGTP insertion and 8-fold more efficient than opposite dA (Table 2).

**Table 2.**
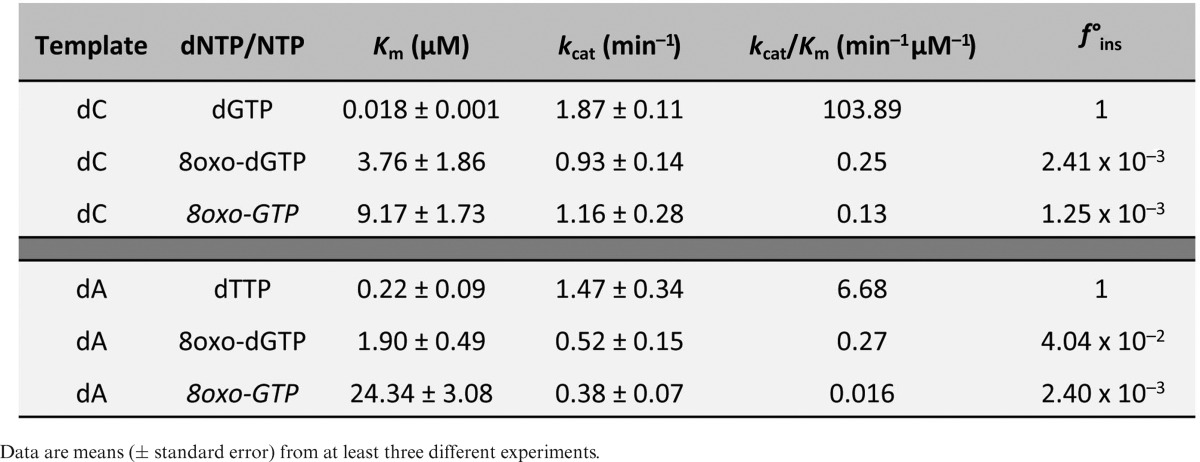
Steady-state kinetic parameters of 8oxo-dGTP and 8oxo-GTP incorporation by purified S*p*Pol4

Once we determined that they are valid substrates, an important question is how these oxidized nucleotides compete with the cognate normal substrates (dGTP/GTP; dTTP/UTP). Remarkably, when copying a template dC, *Sp*Pol4 incorporated undamaged dGTP/GTP with much higher efficiency than 8oxo-dGTP and 8oxo-GTP (Figure 7C). Furthermore, a detailed kinetic analysis demonstrated that *Sp*Pol4 showed low affinity for 8oxo-dGTP and 8oxo-GTP, being incorporated opposite dC 416-fold and 800-fold less efficiently than dGTP (Table 2). When copying a template dA, *Sp*Pol4 also preferred the undamaged nucleotides dTTP and UTP (Figure 7D), being the incorporation of 8oxo-dGTP and 8oxo-GTP 25-fold and 418-fold less efficient than dTTP, respectively (Table 2). Therefore, we can conclude that 8oxo-dGTP and 8oxo-GTP are inefficient substrates for *Sp*Pol4.

### Specificity of the incorporation of 8oxo-dGTP and 8oxo-GTP by *Sp*Pol4 in *S. pombe* cell extracts

**Figure 8.**
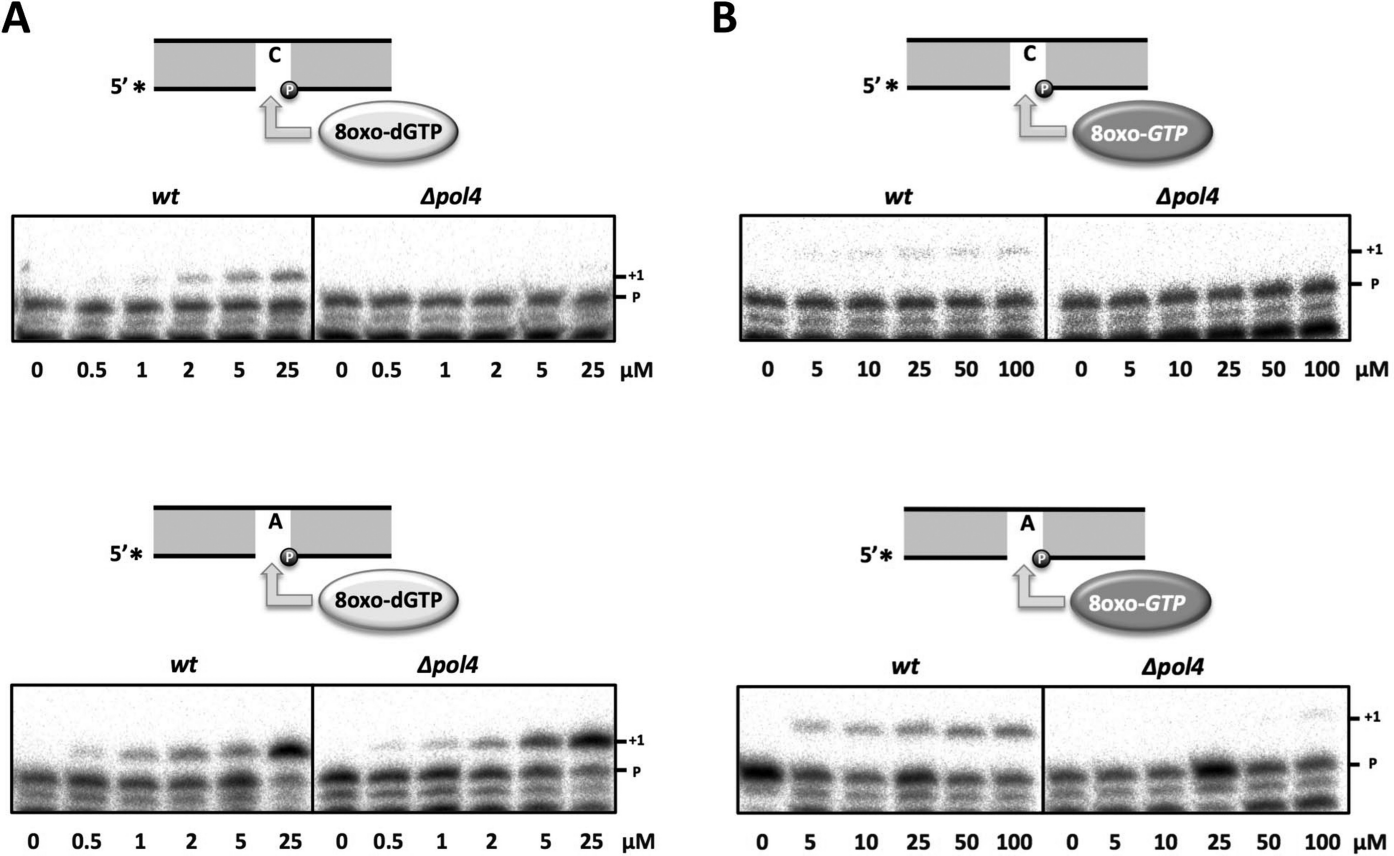
Analysis of 8oxo-dGTP and 8oxo-GTP incorporation by cell extracts. (**A**) Wild-type and *Δpol4* G1 cell extracts (20 μg) were incubated with 1nt-gapped molecules with either a dC or dA template (2.5 nM) and the indicated concentrations of 8oxo-dGTP. After incubation at 30°C for 20 min, primer extension was analysed as described in the ‘Materials and Methods’ section. (**B**) Gap-filling reactions performed as in (A) but using the indicated concentrations 8oxo-GTP.

The incorporation of 8oxo-dGTP and 8oxo-GTP by wild-type and *Δpol4* G1 WCE was evaluated using the 1nt-gapped molecules with either dC or dA in the gap position. The error-free incorporation of 8oxo-dGTP (opposite template dC) was very specific for *Sp*Pol4 activity at any concentration (0.5–25 μM), as incorporation was barely detectable in the *Δpol4* extracts (Figure 8A, top). Conversely, other polymerases present in the WCE incorporated 8oxo-dGTP very efficiently opposite dA, precluding the detection of *Sp*Pol4 possible contribution, if any (Figure 8A, bottom). In agreement with *Sp*Pol4 unique ability to use NTPs, the incorporation of 8oxo-GTP opposite both template dC and template dA was completely *Sp*Pol4-dependent at all the concentrations tested (Figure 8B). It is worthy to note that in contrast to our previous data (Figure 7B), *Sp*Pol4 incorporated 8oxo-GTP more efficiently opposite template dA than opposite dC.

All together, these data demonstrate that *Sp*Pol4 can incorporate 8oxo-GTP and 8oxo-dGTP to the DNA in cell extracts, and that among fission yeast polymerases, *Sp*Pol4 is the only able to incorporate 8oxo-GTP as a valid substrate, both opposite dC and dA. Moreover, incorporation of 8oxo-dGTP opposite dC also appears to be *Sp*Pol4-dependent.

## DISCUSSION

**Figure 9.**
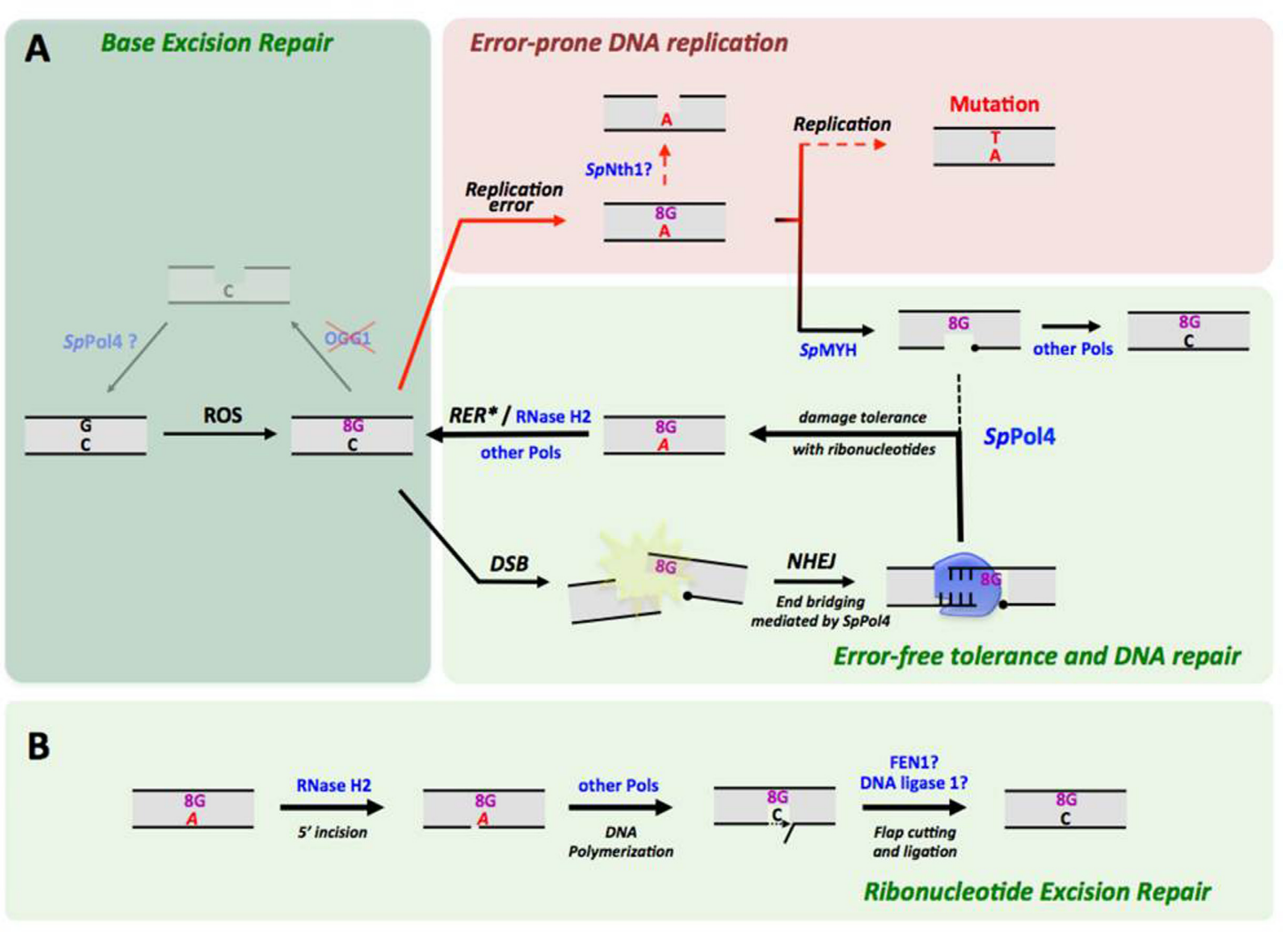
Model for *Sp*Pol4 contribution to *S. pombe* GO system. (**A**) Reactive oxygen species (ROS) can oxidize guanines in the genome of *S. pombe*. The absence of a OGG1 homolog in fission yeast suggests that the premutagenic 8oxodG:dC base pair could be persist in the DNA, prompting the incorporation of dATP during DNA replication. *S. pombe* MutY homolog (*Sp*MYH) can remove the wrong dAMP from the 8oxodG:dAMP base pair, and other polymerases (not *Sp*Pol4) can directly reconstitute a 8oxodG:dCMP base pair. *Sp*Pol4 can tolerate 8oxodG, mainly during NHEJ, using preferably the abundant ATP. That inserted ribonucleotide is then removed by a speciailzed RER mechanism (*the enzymatic reactions are shown in detail in (B)). (**B**) Insertion of ATP by *Sp*Pol4 would not be harmful as RNase H2 can recognize 8oxodG:AMP base pairs to initiate RER, thus triggering the error-free bypass of 8oxodG coupled to strand displacement, and preventing mutagenesis.

One of the most common lesions generated by oxidative stress is 8oxodG. It arises in the DNA upon guanine oxidation (Figure 9A), and is promutagenic due to its ability to mispair with adenine. In mammalian cells, 8oxodG is mainly repaired by BER, in a process initiated by OGG1 (homolog of MutM in *E. coli*). The absence of an OGG1 homologue in *S. pombe* suggests that 8oxodG:dC base pairs could be left unrepaired, thus promoting misincorporation of dATP most likely during DNA replication with a potential impact in mutagenesis (Figure 9A). However, most of these errors should be eliminated by MutY, conserved in fission yeast (*Sp*MYH), by excising the adenine from 8oxodG:dA base pairs (20) (Figure 9A). Coupled to the action of MutY homologs (MYH), a specialized polymerase able to copy 8oxodG in an error-free manner is considered indispensable to avoid mutagenesis, as replicative DNA polymerases display a reduced efficiency and fidelity when tolerating this lesion.

Some DNA polymerases from family X, specialized in performing short polymerization reactions associated to BER and NHEJ, are also well suited to participate in the MutY-dependent conversion of 8oxodG:dA into 8oxodG:dC base pairs, thus having a translesion synthesis (TLS) function. Notably, Polλ (a PolX enzyme) is critical for the 8oxodG repair in humans, acting downstream MYH and incorporating the error-free dCTP almost exclusively (23,24). Here we have shown that the unique PolX in *S. pombe* is not an orthologue of Polλ for performing this specific function, but other endogenous polymerases have this potential.

Conversely, we have demonstrated that unlike human Polλ, *Sp*Pol4 tolerates 8oxodG by preferably incorporating ATP not only during gap-filling, but also during NHEJ (the main function of *Sp*Pol4), and that this ability is not significantly influenced by other repair factors present in the cell extracts. This is the first time showing that ribonucleotides can be used as convenient substrates for tolerating lesions, as relevant as 8oxodG. We have recently shown that ribonucleotides can be valuable substrates for DNA repair, being used by human Polμ during NHEJ, potentiating its fidelity without altering efficiency (30). That is particularly relevant given that their physiological concentrations substantially exceed that of dNTPs (37). Thus, even though we can not discard that *Sp*Pol4 may incorporate ATP following *Sp*MYH activity, *Sp*Pol4 is more likely specialized in tolerating 8oxodG during NHEJ, taking advantage of the highly abundant ATP as a valid substrate to promote efficient repair of the most hazardous lesion for cell viability: DSBs (Figure 9A). *In vivo* analyses demonstrated that *Δpol4* cells are only mildly sensitive to oxidative damage (Supplementary Figure S5), which would support that *Sp*Pol4 could be specialized in the repair of clustered damage, perhaps incorporating ATP during infrequent events caused by 8oxodG persistence and DSB formation.

What is the consequence of this specialization? Remarkably, ATP incorporation opposite 8oxodG leads to the accumulation of two different ‘damages’ (base and sugar) within a single base pair, which is in principle undesirable as ribonucleotides increase the probability of chromosomal strand breakage (46). However, we have demonstrated that the insertion of ATP opposite 8oxodG may not be problematic, as 8oxodG:AMP base pairs were efficiently recognized by RNase H2 to eliminate the AMP (Figure 9B). Strikingly, incision at the 5′-side of AMP was specifically coupled to the incorporation of dCTP opposite 8oxodG, which is *Sp*Pol4-independent (Figure 9B). The identity of the DNA polymerase involved in this ‘mismatch repair’ reaction remains to be determined, and Polδ could be a good candidate given its association to RER in *S. cerevisiae* (44). These data emphasize the relevant role of NTPs for damage tolerance and suggest that ATP incorporation opposite 8oxodG by *Sp*Pol4 could be an intermediate step that triggers a subsequent error-free bypass of the lesion (Figure 9A). *Sp*Pol4 is closely related to human Polμ, which also incorporates ribonucleotides to DNA very efficiently (30), and tolerates 8oxodG preferably in an error-prone manner (47). Therefore, it is tempting to speculate that this mechanism may be conserved in human cells during NHEJ coupled to 8oxodG tolerance, and that it may involve a first step of Polμ-mediated ATP incorporation, followed by RER and Polλ-mediated insertion of dCTP. This would be in agreement with the important role of RNase H2 in maintaining genomic stability in mammalian cells (48).

Another source of 8oxodG/8oxoG accumulation in the genome is the incorporation of the 8oxo-dGTP and 8oxo-GTP from the cellular pool of nucleotides, which could be a relevant scenario in *S. pombe*, as homologs for MutT/MTH1 have not been identified. Our results using fission yeast extracts demonstrate that the endogenous DNA polymerases of *S. pombe* are able to misincorporate 8oxo-dGTP opposite dA, but *Sp*Pol4 contribution was undetectable. Accordingly, the only enzyme capable of removing an incorporated 8oxodG in *S. pombe, Sp*NTH1, operates when the lesion is paired to dA (49), as a specialized mismatch repair probably associated to DNA replication. Remarkably, *Sp*NTH1 activity could interfere with *Sp*MYH, removing a templating 8oxodG from 8oxodG:dA mispairs, but this would be inconvenient and lead directly to mutagenesis (Figure 9A). *Sp*Pol4 could incorporate 8oxo-dGTP/8oxo-GTP during *in vitro* DNA polymerization and was the only polymerase present in extracts capable of incorporating 8oxo-dGTP opposite dC and 8oxo-GTP opposite either dA or dC. However, these oxidized nucleotides are incorporated very inefficiently by SpPol4 in comparison with undamaged nucleotides, requiring a significantly higher concentration than dTTP/UTP and dGTP/GTP for similar efficiency *in vitro*. 8oxo-dGTP concentration is relevant in mammalian mitochondria, which are exposed to high levels of oxidative stress, as it matches dTTP concentration in some tissues, but being only a fraction of the dGTP pool (50). 8oxo-dGTP/8oxo-GTP levels have not been determined in *S. pombe* but are likely to be significant, possibly resembling the situation of mammalian mitochondria; however, the *Sp*Pol4 marked preference for undamaged nucleotides suggests that in a physiological context, the oxidized nucleotides will be probably outcompeted by dGTP/GTP and dTTP/UTP. These results contrast with *Sp*Pol4 ability to tolerate 8oxodG as efficiently as undamaged DNA, and suggests that its active site has been adapted to efficiently tolerate this lesion in a template, but to avoid its incorporation as a dNTP/NTP, which is in line with the absence of a MutT homolog.

A recent study of *Thermus thermophillus* PolX (*Tth*PolX) suggested that the absence of an specific asparagine, relatively conserved among PolXs, could be an adaptation of the active site to avoid 8oxo-dGTP incorporation opposite dA in organisms where the GO system does not sanitize this oxidized nucleotide, although it hampers pyrimidine incorporation (51). This residue is Asn^279^ in Polβ and its mutation to alanine was shown to revert Polβ's preference for incorporation of 8oxo-dGTP opposite template dA, favouring the template dC (52). That residue is absent in *Sp*Pol4, which is in agreement with its preference for purines over pyrimidines (26), and further suggests and adaptation to avoid 8oxo-dGTP/8oxo-GTP accumulation in the genome.

In conclusion, our data suggest that *Sp*Pol4 takes part in the tolerance of 8oxodG in *S. pombe*, most likely during NHEJ. *Sp*Pol4 incorporates preferably ATP opposite 8oxodG, but this error-prone event is just an intermediate step that recruits RNase H2 and other RER enzymes that eliminate the ribonucleotide and triggers dCTP incorporation. Possibly, this could be a conserved mechanism in higher eukaryotes where ribonucleotides could be useful for damage tolerance.

## SUPPLEMENTARY DATA

Supplementary Data are available at NAR Online.

SUPPLEMENTARY DATA
