# Supplementary material for: ATP insertion opposite 8-oxo-deoxyguanosine by Pol4 mediates error-free tolerance in Schizosaccharomyces pombe
Source: Nucleic Acids Res. 2014 Aug 8;42(15):9821–37. doi: 10.1093/nar/gku711 (PMC4150805; doi:10.1093/nar/gku711)
Supplement: SUPPLEMENTARY DATA [file supp_42_15_9821__index.html]

ATP insertion opposite 8-oxo-deoxyguanosine by Pol4 mediates error-free tolerance in Schizosaccharomyces pombe — ATP insertion opposite 8-oxo-deoxyguanosine by Pol4 mediates error-free tolerance in Schizosaccharomyces pombe — SUPPLEMENTARY DATA 

# ATP insertion opposite 8-oxo-deoxyguanosine by Pol4 mediates error-free tolerance in *Schizosaccharomyces pombe*

## SUPPLEMENTARY DATA

**Files in this Data Supplement:**

- SUPPLEMENTARY DATA
