# Supplementary material for: ATP insertion opposite 8-oxo-deoxyguanosine by Pol4 mediates error-free tolerance in Schizosaccharomyces pombe
Source: Nucleic Acids Res. 2014 Aug 8;42(15):9821–37. doi: 10.1093/nar/gku711 (PMC4150805; doi:10.1093/nar/gku711)
Supplement: SUPPLEMENTARY DATA [file supp_gku711_nar-00547-d-2014-File013.pdf]

Supplementary Figure 1. Extension products generated by purified *Sp*Pol4 or WCE during gap-filling opposite 8oxodG. Purified *Sp*Pol4 and other polymerases present in the cell extracts can incorporate two subsequent dATPs when the 8oxodG-containing DNA gap was provided; the first incorporation occurs opposite 8oxodG, and the second is directed by the adjacent dT, thus requiring strand displacement. Purified *Sp*Pol4 can tolerate 8oxodG incorporating only one dCTP, but other polymerases present in the cell extracts can incorporate dCTP twice, both opposite 8oxodG, and the second after realigning the primer one position backwards. *Sp*Pol4 was inefficient extending ribonucleotides, and produced mainly +1 extension products using either ATP or CTP as substrates. Given that the WCE could degrade the primer terminus, a 1 nt-deleted primer could be extended using CTP, generating a band (denoted as +1*, and detectable in Figure 3C) with slightly slower migration than the original primer.

Supplementary Figure 2. Incorporation of ATP opposite 8oxodG by *Sp*Pol4 in competition with physiological concentration of nucleotides. GST-*Sp*Pol4 (35 nM) was incubated with the 8oxodG-containing DNA gap, and serial dilutions of ATP (3000, 300, 30 or 3 μM) and physiological concentrations of dCTP (14 μM), dATP (16 μM) and CTP (500 μM) were added to the reaction as indicated. **Panel 1**: *Sp*Pol4 was able to insert ATP opposite 8oxodG even at the lowest concentration tested (3 μM; see panels 2-5). **Panel 2**: *Sp*Pol4 could not incorporate dCTP when ATP was provided either at 3000 μM, at 300 μM and even at 30 μM, but it was incorporated when ATP was either 1000-fold reduced to 3 μM or was not added (lanes 6-10).

Panel 3: In agreement with our previous data, a physiological concentration of dATP (16 μM) further added to the experiment precluded any dCTP insertion, as the +1 band observed corresponds to the mobility of a primer extended (+1) with dA (lanes 13-15, and panel 4 lanes 18-20). Moreover, a 10-fold reduction of the physiological concentration of ATP (300 μM) was sufficient to outcompete completely dATP (lane 12); however, *Sp*Pol4 could efficiently incorporate dATP with 30 μM ATP (lanes 13), and outcompete 3 μM ATP, on the basis of the slightly different mobility between the (2xdA)+2 *versus* (A)+1 products (lane 14; the origin of this +2 product has been described in Fig. S1). **Panel 4**: Addition of a physiological CTP concentration (500 μM) can compete ATP (note the different mobility of the (A)+1 and (C)+1 products), but only when ATP concentration is at least 100-fold reduced (30 μM; panel 4, see lanes 18-20). The slowest migrating band appearing in panels 2-4, denoted as (dA/dC+A)+2, corresponds to a first extension of the primer using either dA or dC, and a subsequent extension using ATP, mediated through strand-displacement and mimicking the described for two subsequent dATPs incorporations (Fig. S1)

All together, these data indicate that under physiological conditions *Sp*Pol4 will tolerate 8oxodG incorporating ATP almost exclusively, as ribonucleotides are the most abundant substrates and that even CTP can not outcompete ATP when both are provided at physiological concentrations.

Supplementary Figure 3. *Sp*Pol4 incorporates dNTPs and NTPs with similar efficiency during NHEJ *in vitro*. For the analysis of NHEJ *in vitro* by *Sp*Pol4 we used two different dsDNA molecules with 3'-protruding, partially complementary ends. After microsynapsis of the two ends, these molecules would form two 1 nt-gaps, adjacent to

a connection of 4 bps of complementarity (see schemes) that require nucleotide insertion, and further ligation for full repair. (A) NHEJ by GST-*Sp*Pol4 (200 nM) using the labelled end acting as primer (5 nM; light grey), the cold end (12,5 nM; dark grey) providing a templating dG at the gap, and the indicated amounts of either dCTP or CTP. After 60 min of incubation at 30°C, the samples were processed as described in Materials and Methods. (B) NHEJ involving a templating 8oxodG, performed as in (A) but using the indicated amounts of dCTP and dATP.

Supplementary Figure 4. Western-blot analysis of GST-*Sp*Pol4 over-expression. GST-tagged *Sp*Pol4 and control GST-over-expressing *S. pombe* cell extracts (50 µg) were resolved by SDS-polyacrylamide gel electrophoresis. Over-expression of the proteins was demonstrated by western-blot analysis using anti-GST antibodies.

Supplementary Figure 5. *Δpol4* cells are moderately sensitive to H_2O_2. To evaluate whether *pol4* (*h^- Δpol4:hphMX)* or *rnh201* (*h^- Δrnh201:kanMX) strains (obtained in this work)* are more sensitive to oxidative damage than a wild-type (*h^- 972*) strain, cells were grown to mid-logarithmic phase and subsequently treated with 0, 1, 5 and 10 mM of H_2O_2 for 3h at 30 °C. After treatment, 500 cells were plated in YES medium and incubated for 3 days at 30 °C. Colonies were counted and viability was calculated relative to the 0 mM plate. Data with standard deviations from two independent experiments are presented.

The experiment shows that *Δpol4* cells are sensitive to H_2O_2, but this phenotype is not pronounced. Moreover, *rnh201* deletion did not sensitize cells to oxidative damage, which was somewhat expected, since *rnh201* deletion in budding yeast has not been associated with any clear phenotype and does not sensitize cells to hydroxyurea (52); remarkably, this study also showed that the *rnh201* deletion was problematic only when vast ribonucleotide insertion was provoked by mutating the sugar steric gate of the replicative polymerase Pol2. We suggest that the accumulation of ribonucleotides by *Sp*Pol4 during 8oxodG tolerance is probably not sufficient to drive cell death if *rnh201* is depleted.

Likewise, the mild sensitivity caused by *pol4* deletion is also not surprising because we expect *Sp*Pol4 role in tolerating 8oxodG to be associated primarily with clustered damage, after 8oxodG persistence indirectly leads to DSBs. Therefore, we suggest that there are probably other pathways of 8oxodG tolerance in *S. pombe* that are compensating for the lack of *pol4*. This is in line with the fact that depletion of relevant genes for 8oxodG tolerance in mammals causes mild phenotypes, as for instance the case of *ogg1* deletion in mice (53).

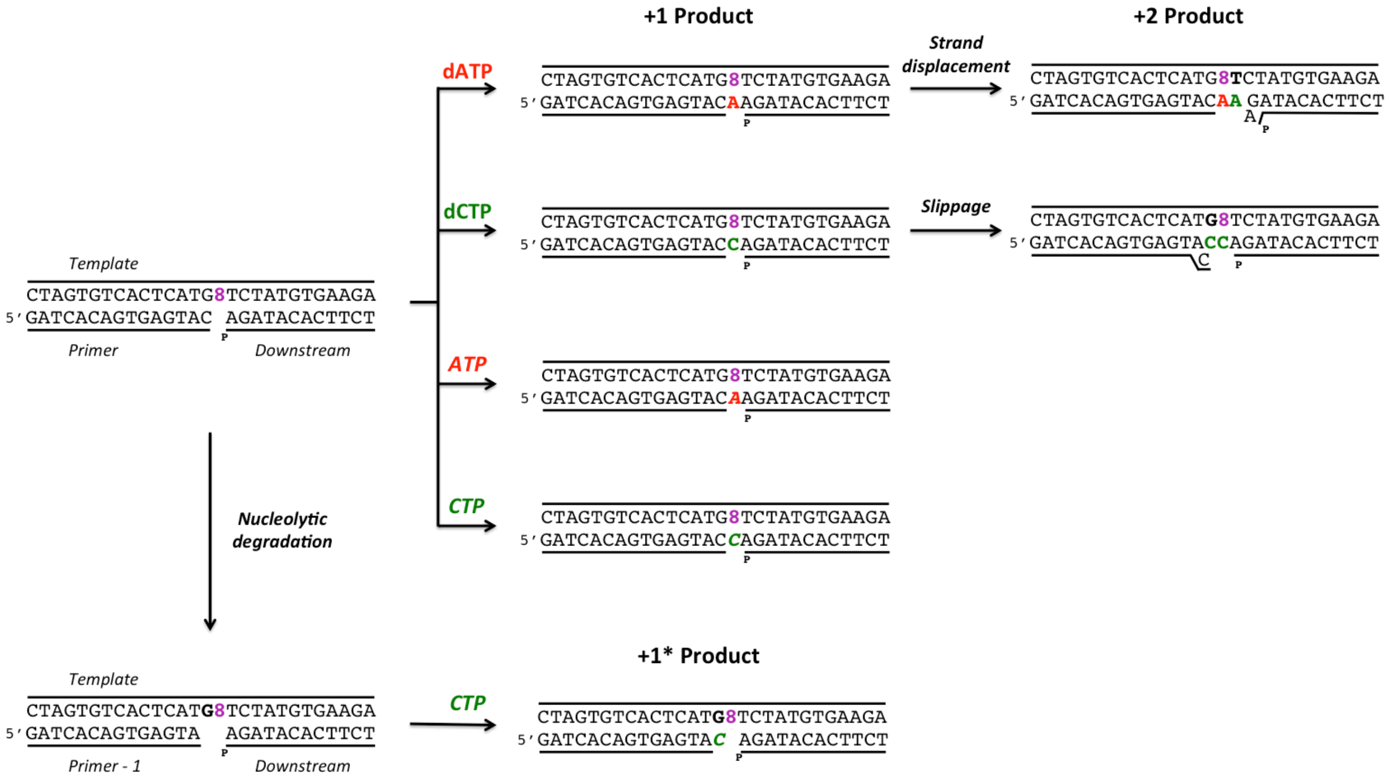

Figure S1 Sastre-Moreno et al.

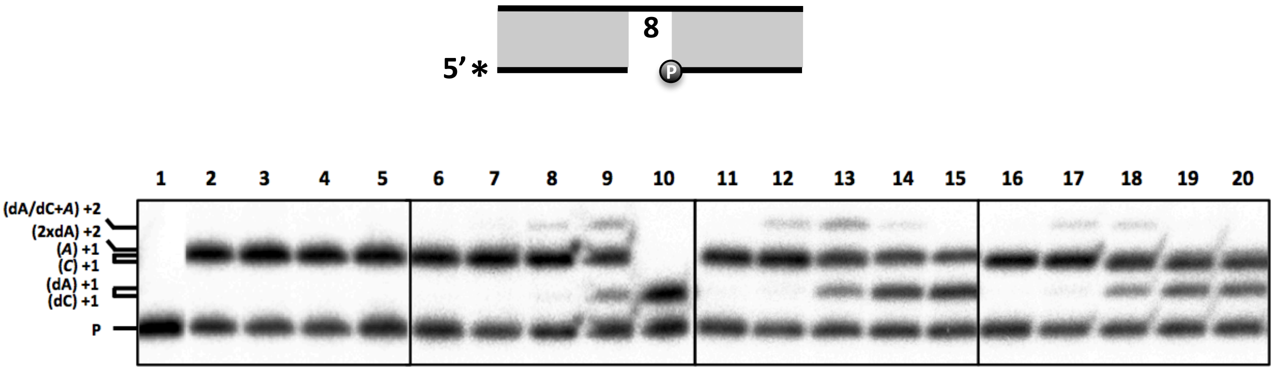

	1	2	3	4	5	6	7	8	9	10	11	12	13	14	15	16	17	18	19	20
ATP (µM)	-	3000	300	30	3	3000	300	30	3	0	3000	300	30	3	0	3000	300	30	3	0
CTP (µM)	-	-	-	-	-	-	-	-	-	-	-	-	-	-	-	500	500	500	500	500
dATP (µM)	-	-	-	-	-	-	-	-	-	-	16	16	16	16	16	16	16	16	16	16
dCTP (µM)	-	-	-	-	-	14	14	14	14	14	14	14	14	14	14	14	14	14	14	14

Figure S2 Sastre-Moreno et al.

A

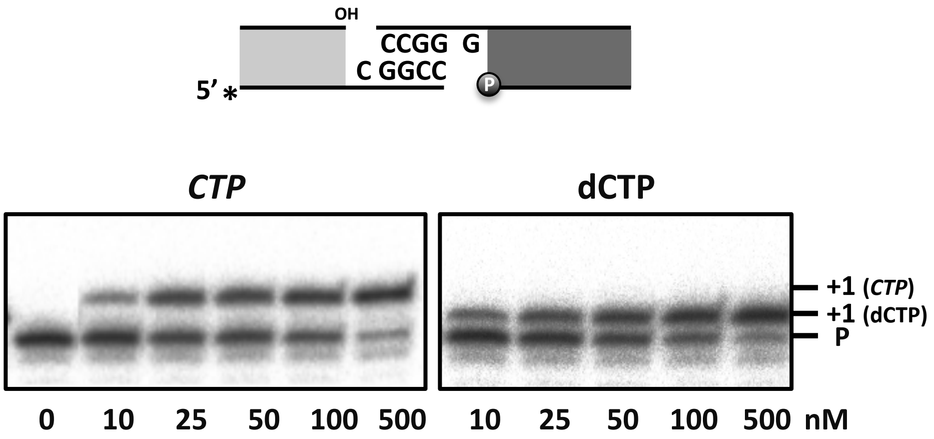

B

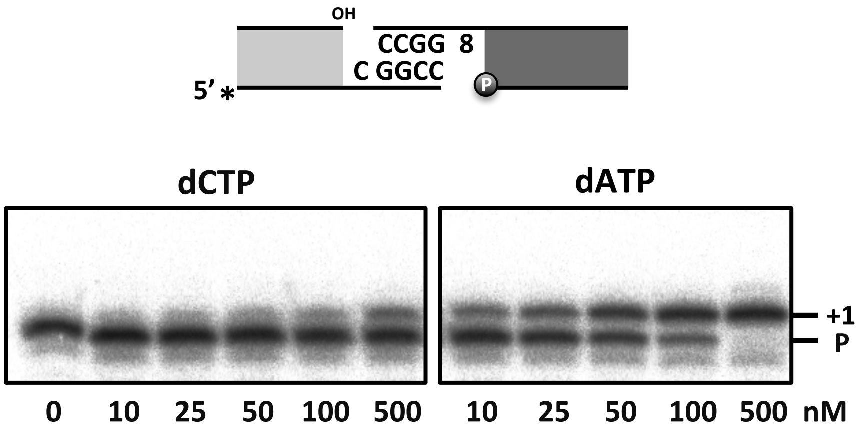

Figure S3 Sastre-Moreno et al.

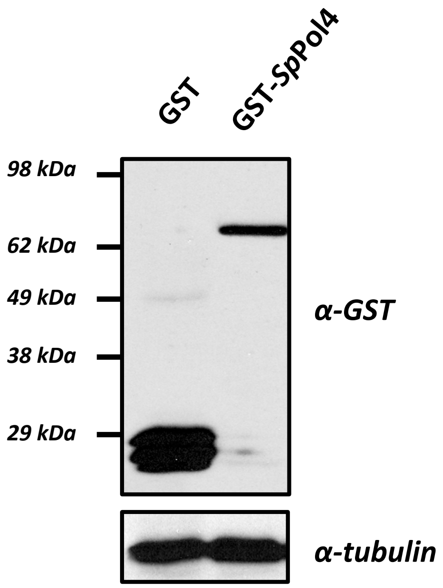

Figure S4 Sastre-Moreno et al.

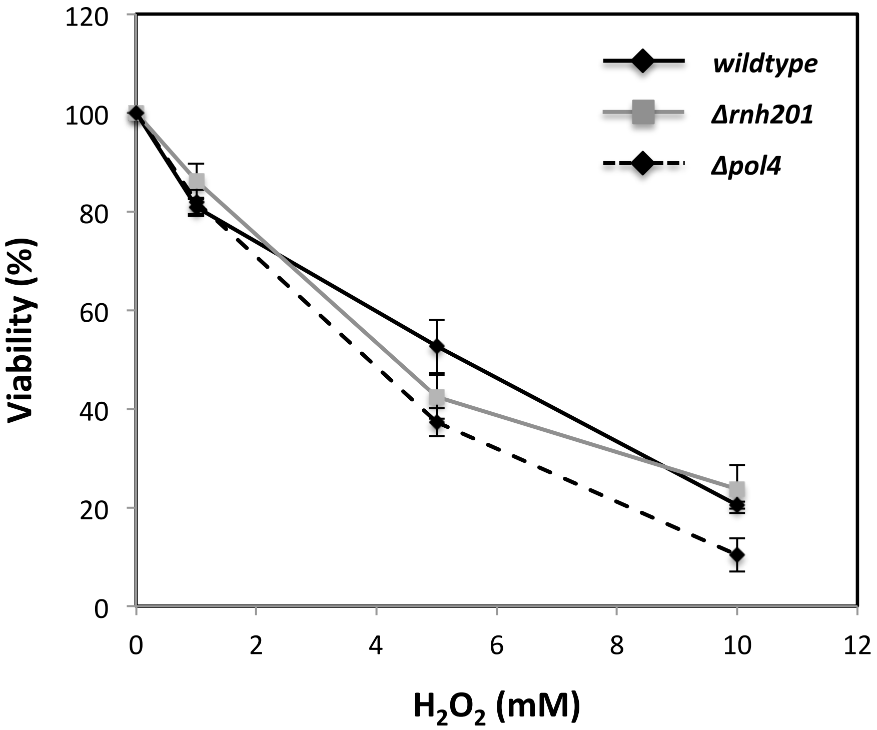

Figure S5 Sastre-Moreno et al.
